# Trained Immunity and Persistent Myeloid Responsiveness in MASH

**DOI:** 10.3390/cells15212022

**Published:** 2026-11-05

**Authors:** Liyuan Fu, Rui Guo, Qingyu Zhang, Qichun Zhang

**Affiliations:** 1 School of Chinese Medicine, Nanjing University of Chinese Medicine, Nanjing 210023, China; 2 School of Medicine, Nanjing University of Chinese Medicine, Nanjing 210023, China; 3 Jiangsu Key Laboratory for Pharmacology and Safety Evaluation of Chinese Materia Medica, School of Pharmacy, Nanjing University of Chinese Medicine, Nanjing 210023, China

**Keywords:** metabolic dysfunction-associated steatohepatitis, trained immunity, hepatic macrophages, immunometabolic reprogramming, epigenetic memory

## Abstract

Metabolic dysfunction-associated steatohepatitis (MASH) involves hepatocellular injury, myeloid activation, and fibrotic remodeling that may resolve unevenly following metabolic improvement. Whether previous injury leaves a functionally consequential innate immune imprint remains unresolved. This review evaluates trained immunity as a candidate mechanism linking exposure history to persistent hepatic myeloid responsiveness. We distinguish continued activation by lipotoxic and stromal signals from responses retained after stimulus withdrawal, macrophage replacement, and hematopoietic progenitor programming. Evidence from Kupffer cells, monocyte-derived macrophages, and lipid- or scar-associated populations reveals divergent inflammatory, efferocytosis, and matrix remodeling functions. Receptor-dependent lipid handling intersects with glycolysis, citrate-derived acetyl-CoA, serine and one-carbon metabolism, NAD^+^ availability and histone methylation, acetylation, and lactylation. These pathways explain how metabolic inputs regulate inflammatory competence, but their alteration during active MASH does not establish immune memory. Rested monocyte training models provide stronger temporal evidence than most disease-specific studies, whereas protective TREM2-dependent responses caution against indiscriminate suppression of disease-associated macrophages. Establishing a contribution to MASH recurrence requires lineage-resolved investigations spanning injury, regression, and rechallenge, with interventions separating memory establishment, maintenance, and effector output. If exposure-dependent myeloid memory contributes causally to MASH recurrence, targeting maladaptive responsiveness could complement injury control while preserving hepatic repair.

## 1. Introduction

Metabolic dysfunction-associated steatohepatitis (MASH) is characterized by hepatocellular ballooning and lobular inflammation in a steatotic liver, with fibrosis developing through repeated injury and wound-healing responses [1]. These lesions describe distinct biological processes: hepatocyte lipid accumulation, hepatocellular injury, inflammatory cell recruitment, and extracellular matrix deposition need not resolve together. Their clinical consequences also differ. In a prospective cohort of 1773 adults with biopsy-characterized nonalcoholic fatty liver disease (NAFLD), advanced fibrosis and cirrhosis were associated with increased liver-related complications and mortality [2]. Understanding inflammatory activity, therefore, requires more than documenting hepatic fat or fibrosis stage; it requires identifying how hepatocyte injury engages immune cells, how those responses influence fibrogenesis and repair, and whether their subsequent behavior depends on earlier exposure.

Hepatic macrophages are central to understanding whether previous liver injury alters hepatic responses to subsequent insults, as their composition and functions change during the development and resolution of steatohepatitis. Daemen et al. showed that high-fat, high-sucrose feeding reduced mature TIM4-positive Kupffer cells while increasing monocyte-derived macrophages, including populations expressing Cx3cr1/Ccr2 or lipid-associated macrophage markers [3]. Importantly, Ccr2 deficiency prevented formation of hepatic crown-like structures and increased fibrosis, demonstrating that recruited macrophages can contribute to damage containment rather than uniformly promote injury. During regression, Ganguly et al. found that lipid-associated macrophages retained TREM2 expression and acquired restorative functions; comparison of foz/foz mice with and without Trem2 linked this receptor to phagocytosis, lipid handling, and collagen degradation [4]. These experiments establish a functional distinction between macrophage accumulation and pathogenic inflammation. They also highlight the importance of tracing the fate of previously exposed cells: a population present during active disease may subsequently support resolution, remain inflammatory, or be replaced by newly recruited cells.

Clinically, an important distinction is whether metabolic improvement is accompanied by complete inflammatory resolution. In the prospective Lille bariatric cohort, mean body mass index fell from 49.3 to 37.4 kg/m^2^ one year after surgery, alongside reductions in aminotransferases and insulin resistance [5]. Nevertheless, lobular inflammation improved in 67.1% of assessed samples, and steatohepatitis persisted in a minority of patients. This demonstrates heterogeneous histological improvement despite substantial metabolic benefit at the cohort level; it does not establish inflammation independent of residual metabolic injury. Indeed, persistent steatohepatitis was associated with less weight loss. Longer follow-up provides an important qualification: steatohepatitis resolution was observed in 84% of patients with available five-year biopsies, with no significant recurrence between years one and five [6]. Sustained metabolic improvement can, therefore, accompany durable remission. Contemporary treatment studies extend the opportunity to study incomplete resolution: at week 72 of the ESSENCE trial, semaglutide achieved steatohepatitis resolution without worsening fibrosis in 62.9% of patients, compared with 34.3% receiving placebo [7]. Clinical definitions also matter: in the earlier semaglutide trial, NASH resolution required disappearance of ballooning but allowed mild residual lobular inflammation, scored 0 or 1 [8]. Thus, histological resolution and complete absence of inflammation are not interchangeable; the endpoint definition alone does not quantify how often residual inflammation remains. Such response distributions identify a clinically relevant problem but do not show which immune mechanisms distinguish responders from patients with residual activity.

The liver-directed intervention in MAESTRO-NASH provides a complementary comparison: among 966 participants in the primary analysis, 52-week NASH resolution without worsening fibrosis occurred in 25.9% and 29.9% with daily resmetirom at 80 and 100 mg, respectively, versus 9.7% with placebo [9]. These response rates establish efficacy and heterogeneity but do not identify retained myeloid responsiveness as the cause of nonresponse. Residual hepatocyte injury, incomplete metabolic improvement, and continuing tissue-derived stimulation must be distinguished from an exposure-dependent change in myeloid function. Failure to achieve histological resolution at a treatment endpoint must also be distinguished from recurrence after documented remission. The clinical question is, therefore, whether previous disease alters subsequent inflammatory responsiveness beyond the effects of current injury.

Persistent tissue inflammation can also be obscured by improving systemic measures. In a small longitudinal study of bariatric surgery, Hagman et al. assessed blood and subcutaneous adipose tissue before surgery and at one and six to twelve months [10]. At the later assessment, weight loss and improved glucose tolerance and insulin sensitivity coincided with reduced circulating C-reactive protein, whereas adipose leukocyte infiltration remained elevated and proinflammatory cytokine transcripts were unchanged. This extrahepatic observation supports examining tissue inflammation directly; it cannot establish persistent hepatic inflammation. Evidence concerning recurrence requires similar anatomical precision. Nakanishi et al. reported recurrent ultrasonographic fatty liver in 49.0% of men who had previously achieved remission, associated with weight regain and lack of exercise [11]. The measured event was recurrent steatosis, not biopsy-confirmed inflammatory MASH. Together, these studies motivate distinguishing residual hepatic inflammation from recurrent lipid accumulation and asking whether previous disease increases inflammatory responsiveness to renewed metabolic stress beyond the effect of the renewed exposure itself.

**Figure 1:**
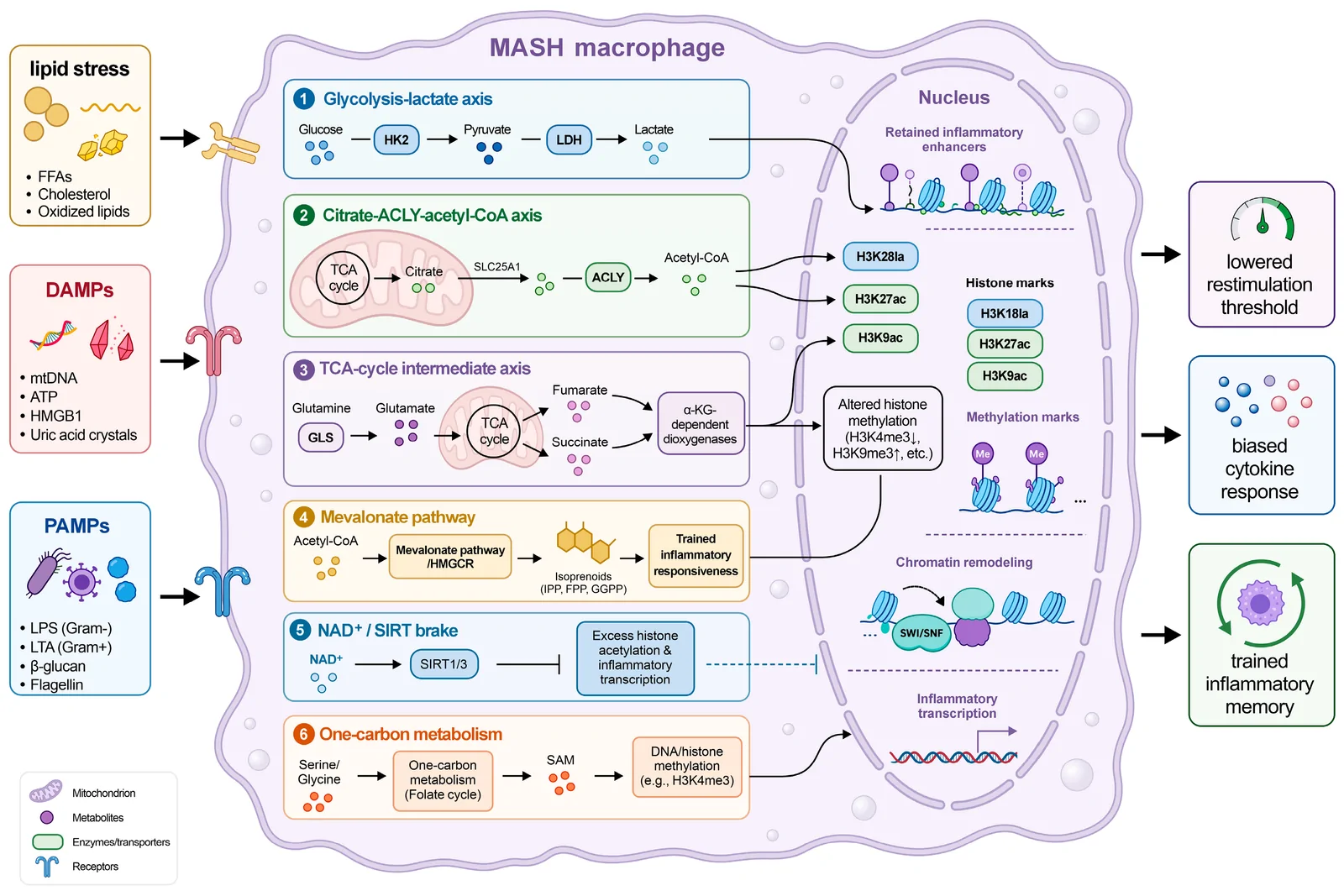
Acute activation and candidate mechanisms of persistent myeloid inflammation in MASH. Lipid stress, hepatocyte-derived damage signals, and gut-derived microbial products activate hepatic myeloid cells. Repeated exposure may sustain inflammation through ongoing injury, fibrotic niche instruction, and retained cellular responsiveness. Memory retention, progenitor reprogramming, and therapeutic normalization are proposed components of the framework, rather than established sequences in MASH. DAMPs, damage-associated molecular patterns; PAMPs, pathogen-associated molecular patterns; HSCs, hepatic stellate cells.

Trained immunity provides an experimentally grounded explanation for how earlier exposure might influence a later inflammatory response. Initial stimulation can alter innate cell metabolism and chromatin regulation so that subsequent stimulation elicits enhanced responses after an intervening resting period. In human monocyte experiments, Cheng et al. connected β-glucan-induced training to aerobic glycolysis through the Akt-mTOR-HIF-1α pathway; inhibition of these signaling components blocked training [12]. This establishes a mechanistic relationship between metabolic adaptation and later immune function, rather than equating increased glycolysis with memory. In MASH, repeated lipotoxic injury and inflammatory stimulation raise the possibility that hepatic macrophages or their hematopoietic precursors retain metabolic and epigenetic changes after the initiating stimuli have diminished. Whether these changes constitute trained immunity requires distinguishing retained cellular responsiveness from persistent hepatocyte injury, continued signals from the hepatic microenvironment, and altered macrophage composition. We propose that exposure-dependent innate immune reprogramming may contribute to incomplete inflammatory resolution and heightened responses to subsequent metabolic or microbial challenges, linking previous disease activity to the subsequent course of MASH. The conceptual distinction between acute activation and candidate persistence mechanisms is summarized in Figure 1.

This narrative review uses a mechanism-oriented selection of studies to examine whether previous MASH exposure alters subsequent myeloid responsiveness. Selection focused on clinical resolution, hepatic macrophage identity and function, exposure withdrawal designs, and causal metabolic or epigenetic regulation. Clinical and experimental MASH studies and human liver atlases were considered alongside comparator training and tolerance models, with disease relevance and temporal–causal evidence evaluated separately. Evidence for continuing injury, population replacement, and protective macrophage functions was included to assess alternative explanations. The review evaluates this mechanistic question rather than providing an exhaustive literature survey or a systematic review.

## 2. Trained Immunity and Myeloid Reprogramming in MASH

The biological significance of previous exposure in MASH depends on what persists as metabolic injury subsides: the initiating signals, the cellular composition of the liver, or the response program of individual myeloid cells. Trained immunity describes an exposure-dependent adaptation in which metabolic and epigenetic reprogramming enhances responses to a subsequent challenge after an intervening period of reduced activation [13]. For this review, evidence that trained immunity contributes to MASH recurrence must address two distinct questions. First, does an identified myeloid population retain altered responsiveness after attenuation of the initiating injury? This requires a recovery interval with assessment of residual stimulation and a standardized secondary challenge, while accounting for basal activity, cell viability, and population composition. Second, does the retained response contribute causally to renewed hepatic injury? This requires comparable secondary exposure and a perturbation that modifies the excess response attributable to prior disease without acting solely through cell depletion, reduced recruitment, or acute suppression during rechallenge. These are proposed criteria for evaluating the evidence; persistent cells, inflammatory signatures, or chromatin alterations alone do not satisfy them. Its experimental foundation includes protection against Candida reinfection in mice lacking functional T and B lymphocytes, together with Dectin-1/Raf-1-dependent reprogramming of human monocytes, linking prior exposure to later innate function [14]. Sterile metabolic exposures extend this principle beyond antimicrobial defense. In Ldlr-deficient mice, four weeks of a Western diet followed by four weeks of chow reduced circulating inflammatory mediators while leaving exaggerated responses to innate stimuli and altered myeloid progenitor regulatory programs; Nlrp3 deficiency prevented major components of this reprogramming [15]. In a complementary diabetes model, macrophages derived from diabetic donors retained altered inflammatory programs when differentiated at physiological glucose, and transfer of diabetic bone marrow into normoglycemic Ldlr-deficient recipients increased atherosclerosis [16]. Dietary withdrawal and transfer into a different metabolic environment make these findings more informative about exposure history than measurements during ongoing hyperlipidemia or hyperglycemia. They establish that systemic metabolic injury can leave durable effects on myeloid function, providing a rationale for examining analogous processes in MASH. Whether the hepatic response is carried by previously exposed Kupffer cells, recruited monocytes, or their bone marrow precursors remains a disease-specific question.

Human vaccination studies link this temporal definition to function. Kleinnijenhuis et al. found increased TNF and IL-1β responses to unrelated microbial stimuli for at least three months after BCG vaccination; NOD2-dependent induction and increased H3K4 trimethylation supplied receptor and regulatory evidence [17]. In a randomized human challenge study, Arts et al. subsequently observed reduced viremia after challenge with an attenuated yellow fever vaccine strain in BCG recipients. Protection correlated with enhanced IL-1β responses rather than the specific IFN-γ response [18]. The first study connects exposure to delayed heterologous cytokine responsiveness, whereas the second adds an organism-level challenge endpoint. Neither identifies a hepatic carrier or a mechanism of MASH relapse.

Previous stimulation can generate divergent forms of myeloid adaptation. Persistent activation denotes continuing stimulus-dependent signaling and effector production; priming prepares a subsequent response without necessarily demonstrating an intervening return towards baseline. Training includes temporal separation between initial activation and an enhanced secondary response, whereas tolerance reduces selected responses to later challenge. Saeed et al. compared primary human monocytes exposed for 24 h to β-glucan, LPS, or culture medium, followed by differentiation and restimulation on day 6 [19]. The common culture interval and secondary assay distinguished conditioning-dependent effects from acute activation, while transcriptomic and epigenomic analyses separated trained and tolerized macrophages from unconditioned differentiation. Ifrim et al. further showed that the identity and concentration of pattern recognition receptor ligands influence whether human monocytes develop enhanced or reduced subsequent cytokine production [20]. Thus, repeated exposure to gut-derived microbial products in MASH cannot be assumed to uniformly increase inflammatory responsiveness. Tolerance is also selective rather than a complete loss of immune competence: Foster et al. identified chromatin-dependent silencing of a class of proinflammatory genes alongside retained or primed inducibility of antimicrobial effectors in LPS-conditioned macrophages [21]. These findings explain how attenuated TNF or IL-6 production can coexist with a substantially reorganized effector repertoire. For hepatic macrophages, assigning a trained or tolerant state requires specifying the conditioning sequence and the function assayed; an inflammatory transcriptomic signature alone does not resolve whether cytokine production, microbial defense, lipid processing, and tissue repair have changed in the same direction.

Metabolic adaptation provides a mechanism through which transient stimulation can influence later responsiveness. Cheng et al. linked β-glucan conditioning of human monocytes to increased glucose consumption and lactate production through Dectin-1-Akt-mTOR-HIF-1α signaling; inhibition during induction prevented the enhanced secondary response, and myeloid HIF-1α deficiency impaired trained protection in a bacterial sepsis model [12]. Arts et al. extended this mechanism to glutaminolysis and fumarate accumulation, showing that fumarate inhibits KDM5 histone demethylases and connects altered intermediary metabolism to the maintenance of activating chromatin features [22]. The mevalonate pathway supplies a distinct regulatory input: pharmacological and genetic experiments identified mevalonate-dependent IGF1-R-mTOR activation and histone remodeling, while statins prevented induction of the trained phenotype [23]. The same study identified constitutive immunological and epigenetic features of training in monocytes from patients with mevalonate kinase deficiency, providing human disease evidence for metabolite-associated recurrent sterile inflammation rather than a demonstration in MASH. Oxidized LDL conditioning also produced sustained cytokine hyperresponsiveness and enhanced foam cell formation in human monocytes, with increased H3K4me3 at inflammatory and scavenger receptor gene promoters six days after initial exposure [24]. These experiments make altered lipid metabolism relevant to both inflammatory output and lipid uptake, while their different stimuli caution against assuming one universal metabolic program. Saeed’s genome-wide profiling mapped conditioning-associated promoter and enhancer configurations, and Ostuni et al. showed that newly activated latent enhancers in differentiated macrophages can retain regulatory changes after stimulation ceases and mediate accelerated, stronger restimulation responses [19,25]. Together, these findings place metabolism and inducible gene regulation within a connected mechanism of persistent adaptation [26]. Perturbation during induction supports a requirement for the implicated pathways, but increased glycolysis or accessible inflammatory loci during active MASH still need to be connected to subsequent function after metabolic injury attenuates.

Exposure-dependent reprogramming can also be modified by a later encounter, making recovery of appropriate responsiveness a distinct therapeutic objective from suppression of basal inflammation. Novakovic et al. exposed human monocytes to LPS for 24 h, introduced β-glucan before a resting interval, and then challenged the differentiated cells again with LPS [27]. β-glucan partially restored cytokine production and active chromatin features at regulatory regions that remained unresponsive in tolerized macrophages. In an experimental human endotoxemia arm, monocytes obtained from 12 volunteers four hours after LPS administration were treated ex vivo with β-glucan, cultured and rechallenged; IL-6 release recovered in nine of twelve donors and TNF release in eight of twelve [27]. The experiment, therefore, connected reversal of an induced state to a secondary functional endpoint across both culture conditioning and an in vivo human exposure. Recovery was incomplete, however, and did not establish restoration of every transcriptional or chromatin feature. The ligand- and dose-dependent responses described by Ifrim and the gene-selective tolerance demonstrated by Foster further indicate that resetting must be evaluated across a defined repertoire of effector functions [20,21]. Applied to MASH, this framework distinguishes a reduction in constitutive inflammatory activity from restoration of a balanced response to renewed hepatocyte injury or microbial exposure; the latter remains to be demonstrated in disease-specific longitudinal studies.

A clinical metabolic comparison found that monocytes from patients with familial hypercholesterolemia retained an enhanced inflammatory phenotype despite cholesterol lowering with statins [28]. This observation distinguishes lowering a circulating lipid exposure from normalization of inducible myeloid function. It supports examining both endpoints after MASH treatment, while leaving unresolved the contribution of residual exposures, continued progenitor output, and liver-specific cellular history.

Hepatic macrophage reprogramming is embedded within tissue signals that establish and subsequently modify resident identity. After Kupffer cell depletion, Bonnardel et al. showed that recruited monocytes acquire Kupffer cell transcription factors through coordinated interactions with hepatocytes, sinusoidal endothelial cells, and hepatic stellate cells: hepatocytes induced ID3, whereas endothelial and stellate cell signals cooperated through NOTCH–BMP to induce LXR-α [29]. Seidman et al. then demonstrated how this regulatory identity is remodeled during diet-induced NASH, identifying partial loss of the Kupffer cell program, induction of Trem2 and Cd9, and convergence between surviving resident cells and recruited macrophages occupying the Kupffer cell niche [30]. ATAC-seq, H3K27ac profiling, and transcription factor occupancy analyses linked this transition to altered LXR regulation and AP-1/EGR-associated networks; ATF3-LXR co-occupancy and P300 recruitment at disease-induced enhancers, together with Clec4f-directed LXR deletion, supported a regulatory mechanism for selected disease-associated genes [30]. These results connect cellular differentiation and transcription factor activity to macrophage heterogeneity more directly than the observation that inflammatory cells accumulate. Yet a shared disease-associated marker does not make all hepatic macrophages equivalent. Remmerie et al. distinguished osteopontin-expressing recruited macrophages from Kupffer cells in fatty liver, while Guilliams et al. integrated single-cell and single-nucleus profiling with spatial RNA and protein mapping to resolve conserved hepatic niches, including lipid-associated macrophages at bile ducts [31,32]. Their combined implication is that ontogeny, local tissue instruction, and spatial position contribute to the apparent inflammatory state. The regulatory changes demonstrated during active disease provide candidate substrates for persistence; their survival through MASH regression and their effects on renewed challenge require longitudinal analysis within the same cellular populations.

Two independent enhancer studies provide a broader basis for this tissue dependence. Gosselin et al. compared brain microglia and peritoneal macrophages, showing that environmental signals selected distinct enhancer programs through secondary transcription factors cooperating with PU.1 [33]. Lavin et al. profiled seven tissue-resident macrophage populations and identified 12,743 macrophage-specific enhancers; transfer into a new tissue environment reprogrammed even differentiated macrophages [34]. Thus, regulatory plasticity can reflect continuing environmental instruction rather than autonomous retention of an earlier inflammatory exposure. The transfer experiments strengthen the causal case for niche control but do not test MASH regression or rechallenge.

Disease regression introduces a further form of persistence: altered macrophage ontogeny that outlasts overt injury. Tran et al. found that impaired embryonically derived Kupffer cell self-renewal and cell loss during NASH allowed Ly6C-positive monocytes to seed the resident compartment; these monocyte-derived Kupffer cells remained after regression while acquiring mature Kupffer cell markers [35]. Their initially immature, proinflammatory phenotype and their persistence after regression concern different stages and do not by themselves establish heightened post-regression reactivity. A second route could sustain exposure history upstream of the liver, through hematopoietic stem and progenitor cells (HSPCs). β-glucan administration expanded myeloid progenitors with IL-1β/GM-CSF-associated signaling and metabolic adaptation, while BCG exposure reprogrammed stem cells to generate macrophages with enhanced protection against tuberculosis [36,37]. Causal support for regulatory persistence came from de Laval et al.: C/EBPβ deletion erased long-term LPS-induced chromatin and gene expression changes in hematopoietic stem cells [38]. Human evidence extends this route beyond mouse models. Sun et al. profiled bone marrow before and 90 days after BCG vaccination or placebo, finding altered stem cell transcription and progenitor chromatin accessibility; accessibility changes associated with KLF/EGR transcription factor sites correlated with IL-1β secretion from donor-paired peripheral blood mononuclear cells [39]. This paired design supports a connection between marrow adaptation and peripheral function, although correlation does not identify which progenitor change causes the cytokine response. In the periodontitis–arthritis axis, Li et al. showed that inflammatory training was transmissible by bone marrow transplantation to naive recipients and increased arthritis severity, with HSPC IL-1 signaling required for induction [40]. Together with the diabetic marrow transfer experiment, these findings show how renewed myeloid populations can carry a prior systemic exposure into a later disease setting [16,40]. For MASH, distinguishing this possibility from persistence of intrahepatic macrophages requires separate measurements of marrow progenitors, incoming monocytes, and resident hepatic lineages.

Persistence must also be interpreted according to functional direction because macrophages retained during regression can facilitate repair. Daemen et al. found that Ccr2 deficiency reduced recruitment-associated hepatic crown-like structures but increased fibrosis in high-fat, high-sucrose-fed mice, indicating that disrupting recruitment can remove a tissue-protective function [3]. Hendrikx et al. combined localization with bone marrow transplantation and functional analyses to show that hematopoietic Trem2 deficiency impaired lipid handling and extracellular matrix remodeling and worsened steatohepatitis and fibrosis [41]. Ganguly et al. examined regression by switching Western diet-fed foz/foz mice, with or without Trem2, to chow after 12 weeks [4]. Eight weeks later, collagen-stained area had fallen by approximately 70% in Trem2-intact mice and 30% in Trem2-deficient mice relative to each genotype’s pre-regression value, alongside TREM2-associated differences in phagocytosis, lipid handling, and collagen degradation [4]. Normalization to the unequal initial fibrosis burdens improves the regression comparison, although constitutive deficiency can influence both the populations that develop and their later functions. De Ponti et al. supplied a complementary distinction between cellular origin and phenotype: resident Kupffer cells could acquire a lipid-associated macrophage-like state, and genetic targeting of resident and recruited populations identified TREM2-dependent clearance of dying cells as a repair function in both compartments across liver injury models [42]. This evidence makes a persisting TREM2-positive signature compatible with beneficial efferocytosis and tissue recovery. It also means that therapeutic suppression of a shared macrophage program could compromise repair even when some inflammatory outputs are reduced. The initiating signals and cellular compartments must, therefore, be resolved before assigning persistent inflammatory or reparative responses to trained immunity (Table 1).

**Table 1:** Experimental designs supporting different forms of persistent myeloid adaptation and their relevance to MASH.

Study and Context	Initiating Exposure and Cell Population	Withdrawal or Recovery	Secondary Challenge or Functional Assay	Principal Finding	Interpretation for MASH Memory
Seidman et al. [30]; experimental NASH	Dietary injury; resident Kupffer cells and recruited hepatic macrophages	Active-disease analyses discussed here	Chromatin profiling, transcription factor occupancy, and Kupffer cells-directed LXR perturbation	Loss of aspects of Kupffer cells identity and redistribution of disease-associated regulatory activity	Establishes regulatory remodeling during injury; post-regression functional memory is not established
Tran et al. [35]; experimental NASH	Dietary injury; embryonic and monocyte-derivedKupffer cells	Disease-regression arm	Cell persistence and acquisition of mature Kupffer cells markers	Monocyte-derived Kupffer cells remain after regression	Establishes persistent changes in ontogeny and composition; does not establish retained inflammatory hyperresponsiveness
Ganguly et al. [4]; MASH regression	Foz/Foz mice after 12 weeks of Western diet; hepatic macrophage populations	Chow switch; regression assessed over subsequent weeks	DQ type I collagen degradation in macrophages isolated four weeks after withdrawal; tissue regression	TREM2 supports restorative macrophage functions	Direct post-withdrawal functional evidence for repair; no demonstration of enhanced inflammatory rechallenge
Lu et al. [43]; experimental MASH	Continued CDAHFD exposure; macrophage F13A1–PKM2 signaling	No withdrawal interval in the intervention discussed	Macrophage-directed F13A1 knockdown initiated at week 6	Reduced inflammation and fibrosis despite persistent hepatic triglyceride accumulation	Supports metabolic control of ongoing injury, not memory resetting
Li et al. [44]; experimental MASH	Continued MCD diet; activated hepatic macrophages	No withdrawal–rechallenge sequence in the evidence discussed	Myeloid Hk2 deletion; HIF-1α inhibition; H3K18la-associated transcription	Disruption of a glycolytic inflammatory feedback program	Disease-specific activation mechanism; post-regression persistence remains unresolved
Christ et al. [15]; systemic metabolic conditioning	Four weeks of a Western diet in Ldlr-deficient mice; myeloid cells and progenitors	Four weeks of chow	Innate restimulation, including LPS; progenitor transcriptional responses	Altered responsiveness persists after circulating inflammation subsides; NLRP3-dependent reprogramming	Strong metabolic comparator; does not establish a hepatic carrier or MASH recurrence
Saeed et al. [19]; training and tolerance	Human monocytes exposed to β-glucan or LPS for 24 h	Five days without additional conditioning stimulus	LPS restimulation on day 6; cytokine and epigenomic analyses	Conditioning produces divergent subsequent functional states	Establishes temporal separation and functional discrimination in a non-MASH system
Keating et al. [45]; lipid conditioning	Human monocytes exposed to 10 μg/mL oxLDL for 24 h	Washing followed by five days of rest	LPS-induced TNF and IL-6; metabolic assays and H3K4me3	Enhanced secondary responses depend on metabolism during conditioning	Supports lipid-induced training under defined conditions; hepatic applicability requires testing
Arts et al. [22]; metabolite-associated training	Human monocytes exposed to monomethyl fumarate for 24 h	Five days without the initiating stimulus	LPS or Pam3Cys challenge; cytokine and chromatin measurements	Fumarate-associated KDM5 inhibition accompanies delayed hyperresponsiveness	Mechanistic benchmark for metabolite–chromatin coupling; not direct MASH evidence
Ziogas et al. [46]; BCG conditioning	Human monocytes exposed to BCG for 24 h	Five days of rest	LPS-induced TNF and IL-6; H3K18la profiling	Retained responsiveness accompanies persistent lactylation-associated regulatory changes	Temporal evidence in a comparator system; corresponding post-regression hepatic measurements are required

## 3. Metabolic and Tissue-Derived Signals Conditioning Hepatic Myeloid Cells

### 3.1. Lipid Delivery, Hepatocyte Injury, and Vesicular Communication

**Figure 2:**
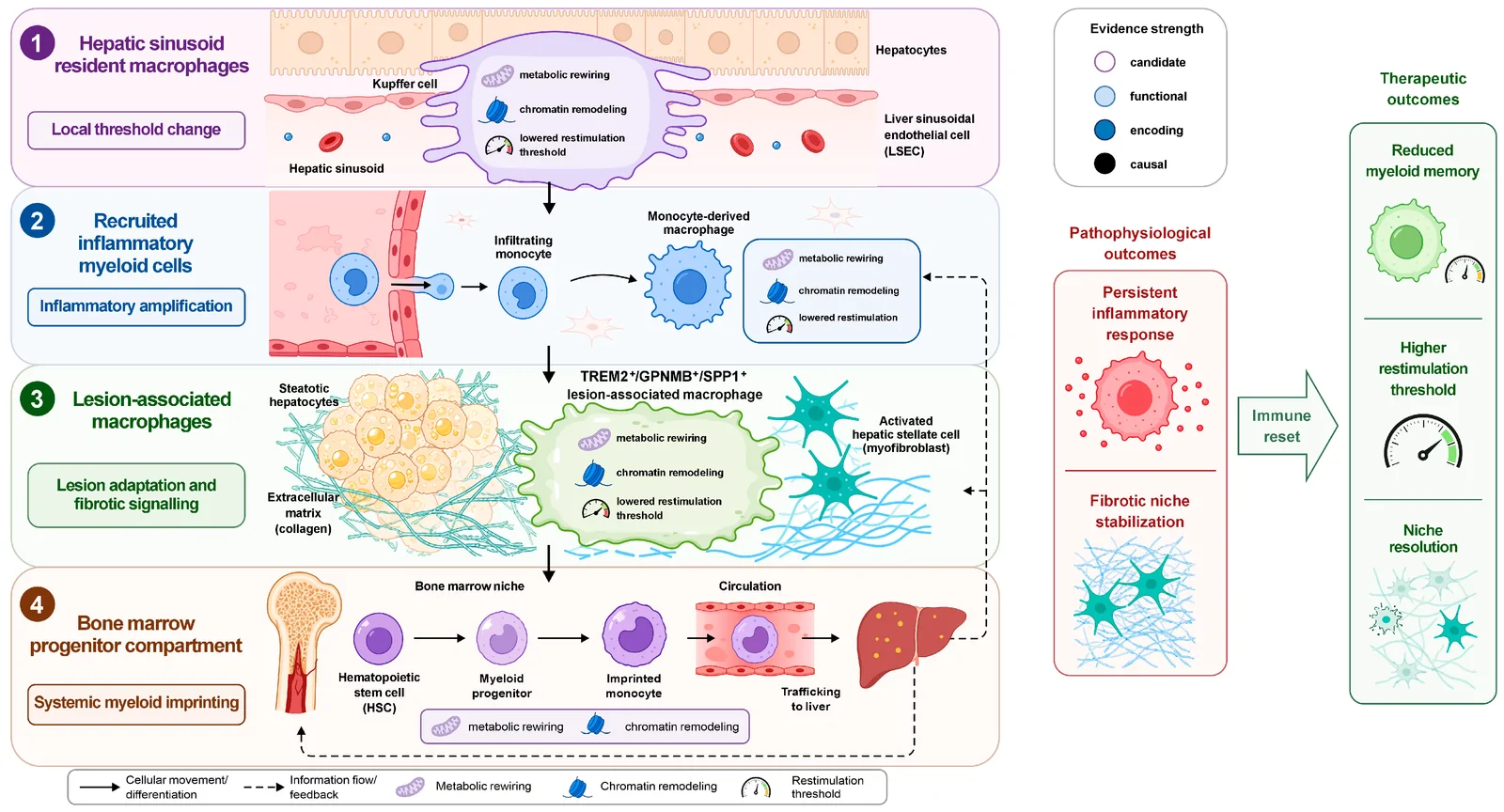
Signals that activate and potentially condition hepatic myeloid cells in MASH. Lipid-derived inputs, hepatocyte injury products, and gut-derived microbial signals contribute to a composite hepatic environment. Receptor signaling, inflammasome activation, and organelle stress connect these exposures to metabolic and chromatin remodeling. Altered secondary responses and persistence require testing after stimulus withdrawal; the schematic does not assign equivalent causal roles to all listed inputs. TLRs, Toll-like receptors; STING, stimulator of interferon genes; ER, endoplasmic reticulum.

The metabolic environment of MASH exposes hepatic myeloid cells to signals generated by both lipid delivery and the hepatocyte response to that delivery. In nine patients with NAFLD studied using stable isotope tracers over four days, Donnelly et al. attributed approximately 59% of hepatic triglyceride fatty acids to circulating non-esterified fatty acids, 26% to de novo lipogenesis, and 15% to dietary sources [47]. These measurements establish the sources sustaining hepatic lipid accumulation; they do not measure the concentrations of individual inflammatory ligands encountered by Kupffer cells. Indeed, human liver lipidomics revealed increased diacylglycerol and triglyceride content in NAFL and NASH without a corresponding increase in total free fatty acids [48]. Thus, an enlarged hepatic triglyceride pool cannot specify the relevant myeloid exposure. The biological question is how lipid composition, hepatocellular processing, and tissue injury determine which signals reach resident Kupffer cells and recruited monocyte-derived macrophages and whether those encounters influence subsequent function. These inputs form a composite environment that can activate and potentially condition hepatic myeloid cells (Figure 2).

Lipid species and their intracellular disposition determine whether hepatocyte lipid uptake produces storage or injury. Malhi et al. showed that saturated fatty acids activated JNK-dependent lipoapoptosis in hepatocyte models; suppression of the BH3-only protein BIM reduced BAX activation and cell death, identifying an injury pathway rather than merely correlating steatosis with inflammation [49]. A complementary in vivo perturbation dissociated triglyceride accumulation from tissue damage. In diabetic db/db mice fed a methionine–choline-deficient diet, DGAT2 antisense oligonucleotides, administered at 25 mg/kg twice weekly for four or eight weeks, reduced steatosis but increased hepatic free fatty acids, oxidative injury, lobular necroinflammation, and fibrosis, despite improved systemic insulin sensitivity [50]. This nutrient-deficient model supports a protective role for esterification under the tested conditions, although it cannot predict the consequences of DGAT2 inhibition across human MASH. Direct macrophage exposure also requires mechanistic precision. Lancaster et al. found that TLR4-dependent priming altered macrophage metabolism and membrane lipid composition sufficiently to permit saturated fatty acid-induced inflammation, while their receptor analyses argued against saturated fatty acids acting as direct TLR4 agonists [51]. Accordingly, lipid-dependent inflammatory competence may reflect prior receptor signaling and intracellular lipid handling rather than direct recognition of palmitate by TLR4.

Hepatocyte-derived extracellular vesicles connect lipid processing to anatomically targeted myeloid exposure, with distinct mechanisms governing vesicle release, macrophage recruitment, and inflammatory activation. Hirsova et al. linked palmitate- or lysophosphatidylcholine-induced vesicle release to hepatocyte DR5-caspase-ROCK1 signaling. The released vesicles carried TRAIL and induced Il1b and Il6 expression in bone marrow-derived macrophages through recipient cell DR5 and RIP1, distinguishing the machinery producing the vesicles from the pathway responding to them [52]. Recruitment was resolved independently: MLK3 inhibition prevented enrichment of CXCL10 in lipotoxic hepatocyte vesicles, and CXCL10-neutralizing antisera inhibited vesicle-induced macrophage chemotaxis [53]. Similarly, IRE1α-XBP1-dependent release of C16:0-ceramide-enriched vesicles promoted chemotaxis through ceramide conversion to sphingosine-1-phosphate; sphingosine kinase and S1P receptor inhibitors interrupted this response [54]. These investigations identify recruitment mechanisms without assigning the recruited cells an inflammatory memory state. Garcia-Martinez et al. subsequently examined vesicles from primary hepatocytes exposed to 800 μM palmitate for 24 h or isolated from mice after 14 weeks of high-fat feeding. Lipidomics showed saturated fatty acid enrichment in vesicles and recipient macrophages; eight-hour exposure increased inflammatory cytokine expression and release, while Tlr4 deletion attenuated signaling in primary Kupffer cells [55]. Conditioned medium from vesicle-exposed macrophages reduced insulin receptor and AKT phosphorylation in hepatocytes, establishing functional feedback from myeloid activation to metabolic dysfunction. Intravenously administered vesicles also localized to CLEC4F-positive Kupffer cells, strengthening the link between the culture experiments and hepatic targeting [55]. In a later study, twice-weekly administration of lipotoxic hepatocyte vesicles for four weeks extended the phenotype to pancreatic inflammation and impaired insulin secretion, implicating macrophage TLR4 in an interorgan response [56]. Collectively, these perturbations support vesicle-mediated communication more convincingly than circulating vesicle abundance alone; the magnitude and cellular distribution of endogenous vesicle exposure during human MASH remain less well defined.

### 3.2. Nucleic Acid Sensing, Inflammasomes, and Gut-Derived Signals

Hepatocellular injury supplies nucleic acid signals whose inflammatory effects depend on their delivery and the recipient signaling compartment. Garcia-Martinez et al. identified hepatocyte-derived circulating microparticles containing mitochondrial DNA in experimental and human NASH; removing these particles reduced plasma TLR9-activating capacity, and deleting Tlr9 in lysozyme-expressing cells reduced high-fat-diet-induced disease [57]. This combination connects a circulating ligand source to a myeloid requirement, although the targeted compartment includes cells beyond Kupffer cells. Yu et al. found that mitochondrial DNA isolated from hepatocytes of high-fat-fed mice induced TNF and IL-6 in cultured Kupffer cells; STING deficiency or NF-κB inhibition attenuated the response, and global STING deficiency reduced disease in high-fat and methionine–choline-deficient models [58]. The culture experiments establish STING-dependent responsiveness, whereas concomitant metabolic changes in globally deficient mice complicate attribution of the in vivo benefit exclusively to Kupffer cells. NLRP3 introduces another distinction: NF-κB-dependent induction of NLRP3 licenses subsequent inflammasome activation, so increased Nlrp3 transcription and caspase-1-dependent cytokine maturation represent different experimental endpoints [59]. Its contribution to NASH also varies across models. In obese Alms1-mutant foz/foz mice with established disease after 16 weeks of an atherogenic diet, MCC950 at 20 mg/kg by gavage five days weekly for eight weeks reduced inflammatory injury and fibrosis [60]. Conversely, six-month high-fat, high-cholesterol feeding produced fibrosing NASH despite global Nlrp3 or Casp1 deletion; MCC950 supplied throughout feeding in drinking water likewise failed to improve histological disease [61]. Differences in genotype, sex, dietary cholesterol, treatment stage, and drug delivery prevent attribution of this discrepancy to one variable. The evidence, therefore, supports context-dependent inflammasome involvement rather than a uniform requirement for NLRP3 in every form of lipid-associated hepatic inflammation.

Hepatocytes can also amplify these encounters before a myeloid cell receives the signal. Csak et al. detected increased caspase-1 activity and mature IL-1β in steatohepatitic mouse livers and showed that palmitate sensitized isolated hepatocytes to LPS-induced IL-1β release. Danger signals released from palmitate-exposed hepatocytes then stimulated inflammasome activity and cytokine production in liver mononuclear cells [62]. This separates hepatocyte sensitization from downstream immune cell activation; it does not demonstrate that either response survives an injury-free rest interval.

Gut-derived microbial products add receptor ligands to this sterile injury environment, but their hepatic delivery cannot be inferred from dysbiosis alone. Henao-Mejia et al. transferred disease susceptibility by cohousing inflammasome-deficient and wild-type mice: the altered microbiota increased portal TLR4- and TLR9-activating signals and aggravated experimental steatohepatitis through TNF-associated inflammation [63]. This finding concerns microbiota-dependent consequences of inflammasome deficiency and should be interpreted separately from inhibition of hepatic inflammasome activity. Mouries et al. resolved a temporal route of delivery in mice fed a diet providing 45% of calories from lard: epithelial ZO-1 loss and mucosal bacterial translocation appeared at 48 h, followed by increased endothelial PV1 and gut vascular leakage at one week [64]. Transfer of microbiota from one-week high-fat-fed donors reproduced vascular barrier disruption, while endothelial β-catenin activation limited hepatic bacterial translocation and protected against subsequent disease; the endothelial intervention was not restricted to the intestine. The inflammatory consequences of microbial DNA recognition were supported by a 22-week choline-deficient amino acid-defined model, in which Tlr9, Myd88, or Il1r deletion reduced disease and CpG-stimulated Kupffer cells produced IL-1β capable of promoting hepatocyte injury and stellate cell fibrogenic responses [65]. However, barrier failure need not initiate the sequence: Luther et al. detected liver injury by day 6 of methionine–choline-deficient feeding, whereas increased intestinal permeability and altered ZO-1 localization became significant at day 10 [66]. Together, these studies support model-dependent entry and amplification of microbial signals, with intestinal permeability potentially preceding or following hepatic injury rather than constituting a universal first event.

### 3.3. Lipid Processing and Scavenger Receptor Signaling

The local organization of injury further determines the duration and biological meaning of macrophage exposure. After six months of high-fat feeding with graded dietary cholesterol, Ioannou et al. observed cholesterol crystallization in hepatocyte lipid droplets and macrophage crown-like structures in the groups developing NASH; NLRP3 and activated-caspase-1 staining localized inflammatory machinery to these lesions [67]. Itoh et al. identified hepatic crown-like structures containing macrophages around injured, lipid-laden hepatocytes in Western diet-fed Mc4r-deficient mice and in human NAFLD, with their abundance associated with fibrosis [68]. These observations identify sites where lipids and dying cell material coexist, but the macrophages occupying such sites can participate in containment and clearance. Spatial proteogenomics placed lipid-associated macrophages near bile ducts in healthy liver and within steatotic regions in obese liver, linking their phenotype to local lipid exposure [32]. Functionally, Ccr2 deficiency prevented accumulation of a recruited CX3CR1/CCR2-expressing macrophage subset and crown-like structure formation yet increased fibrosis, arguing against treating every macrophage aggregate as a purely pathogenic unit [3]. More recent intervention studies reinforce this distinction. In mice maintained on a high-fat, high-carbohydrate diet, vertical sleeve gastrectomy after approximately 14 weeks of feeding improved liver injury relative to pair-fed sham controls; five-week analyses showed altered inflammatory and lysosomal programs in hepatic TREM2-positive macrophages, and global Trem2 deficiency abolished the reparative benefit [69]. Because the deletion was systemic, it does not assign the surgical response exclusively to one hepatic subset. Complementary population-targeted experiments showed that TREM2-dependent clearance by both recruited lipid-associated macrophages and resident Kupffer cells adopting a lipid-associated phenotype supported tissue repair [42]. The relevant conditioning environment, therefore, includes the balance between continued uptake of injured cell material and effective efferocytosis, with macrophage location, ontogeny, and disease stage determining whether the resulting program sustains injury or promotes resolution.

The physical form of cholesterol provides an additional distinction between exposure and its intracellular consequences. Rajamäki et al. showed that cholesterol crystals induced mature IL-1β secretion in human macrophages; NLRP3 silencing abolished this response, whereas cathepsin B inhibition reduced it, implicating lysosomal destabilization [70]. Sheedy et al. then demonstrated that CD36-dependent uptake could convert soluble oxidized LDL into intracellular crystals, coupling ligand internalization to lysosomal disruption and NLRP3 activation [71]. These perturbations identify how macrophage processing generates inflammatory competence rather than merely responding to pre-existing extracellular crystals. Because these experiments concern sterile inflammation outside a MASH withdrawal–rechallenge design, they support a conditioning mechanism, not proof of persistent hepatic memory.

Scavenger receptor experiments further distinguish lipid acquisition from the signaling consequences of lipid composition. In 170 human NAFLD biopsies, Govaere et al. associated macrophage scavenger receptor 1 (MSR1/CD204) expression with foamy macrophages and steatohepatitis; global Msr1 deletion reduced hepatic inflammation and fibrosis after 16 weeks of high-fat, high-cholesterol feeding [72]. In bone marrow-derived and primary hepatic macrophages, MSR1 supported uptake of both palmitate and oleate, whereas saturated fatty acid exposure preferentially induced JNK phosphorylation and Tnf/Il6 expression. Receptor blockade reduced these responses, and JNK inhibition suppressed inflammatory gene induction, linking lipid entry to a defined signaling pathway rather than treating lipid accumulation as an inflammatory endpoint. The intervention results were more selective than the genetic phenotype: two anti-MSR1 doses at weeks 10 and 11 of a 12-week dietary challenge reduced foamy macrophages, Tnf expression, and fibrosis but did not significantly change steatosis grade, ballooning, or lobular inflammation. Human liver slices from two donors exposed for 16 h to 2 mM oleate plus 1 mM palmitate also released less TNF after MSR1 blockade. Concordance across receptor perturbation, kinase inhibition, and human tissue strengthens the lipid–MSR1–JNK mechanism, while the small human experiment and systemic metabolic effects of constitutive deletion limit cell-specific and clinical attribution. Compared with CD36-dependent intracellular crystal formation, this mechanism identifies a different route from lipid handling to inflammatory output [71]. Neither study establishes that receptor-dependent responsiveness remains elevated after lipid withdrawal.

These MASH-associated inputs provide plausible conditioning stimuli, while temporal experiments in other metabolic settings show how exposure can outlast overt stimulation. Seufert et al. exposed bone marrow-derived macrophages to 0.5 mM palmitate with or without 10 μM fumonisin B1 for 12 h, removed the medium, and challenged the cells with 10 ng/mL LPS for 24 h. Ceramide synthase inhibition reduced the amplified TNF, IL-6, and IL-1β response, implicating lipid metabolism in sensitization [73]. Their stronger temporal separation was in vivo: nine daily injections of an ethyl-palmitate formulation followed by seven days without injections increased LPS-induced hypothermia, although the survival difference in that rested cohort was not statistically significant. In Ldlr-deficient mice, four weeks of a Western diet followed by four weeks of chow allowed serum inflammation to subside while enhanced myeloid responses and progenitor reprogramming persisted; Nlrp3 deletion prevented the diet-induced program [15]. Edgar et al. supplied a disease-relevant transfer test: macrophages derived from diabetic mice retained inflammatory characteristics during differentiation at physiological glucose, and bone marrow from diabetic donors increased aortic root atherosclerosis in normoglycemic Ldlr-deficient recipients, accompanied by altered chromatin accessibility and RUNX1-associated regulation [16]. These models establish that metabolic exposure can produce sustained innate functional changes, but they neither identify the hepatic population carrying such changes in MASH nor establish their contribution to relapse. These benchmarks motivate identifying which hepatic lineages are exposed, survive regression, or are replaced during MASH.

## 4. Hepatic Myeloid Populations Sustaining Exposure-Dependent Functional Changes

### 4.1. Kupffer Cell Identity, Replacement, and Recruitment

**Figure 3:**
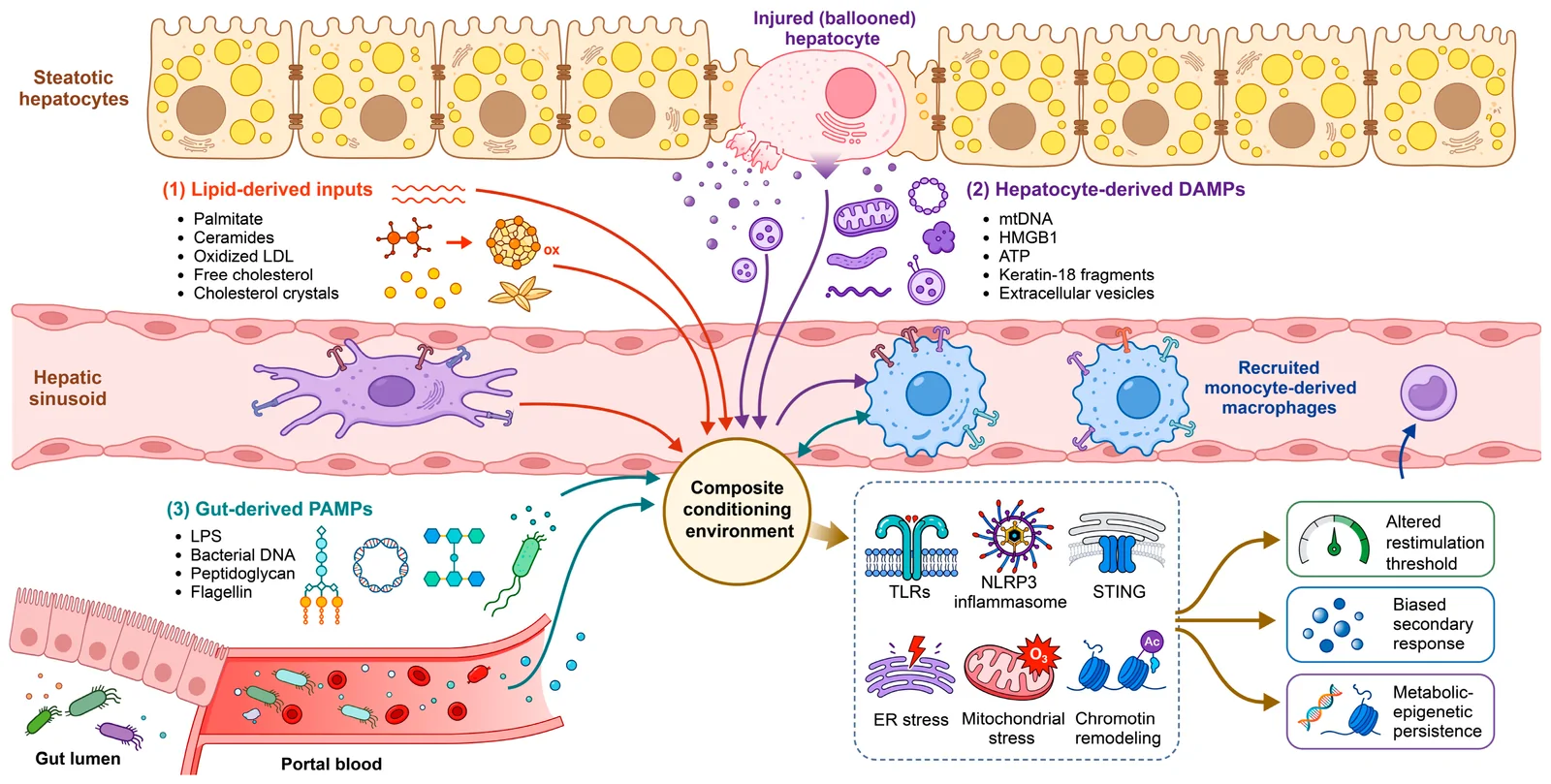
Candidate cellular compartments for exposure-dependent myeloid changes in MASH. Resident Kupffer cells, recruited monocyte-derived macrophages, lesion-associated macrophages, and bone marrow progenitors may contribute through different cellular histories and functions. The evidence key separates MASH-associated states, functional evidence, and cross-context persistence benchmarks. The arrangement does not imply a sequential lineage from Kupffer cells to recruited macrophages. Lesion-associated populations can support injury or repair, and retained responsiveness requires compartment-specific functional validation. HSPCs, hematopoietic stem and progenitor cells; LSECs, liver sinusoidal endothelial cells.

Identifying the cellular carriers of previous metabolic injury requires separating the persistence of individual macrophages from the persistence of a macrophage population or transcriptional state. These distinctions are particularly consequential in the liver, where resident Kupffer cells, monocyte-derived Kupffer cells, and macrophages outside the Kupffer cells niche can coexist despite overlapping surface markers. In a Kupffer cells-depletion model, Scott et al. used diphtheria toxin to create niche availability and showed that circulating monocytes engrafted, progressively acquired the Kupffer cells transcriptional profile, and became long-lived, self-renewing macrophages [74]. Thus, adult residence and self-renewal do not establish embryonic origin. Bonnardel et al. subsequently combined Kupffer cells depletion, analysis of engrafting monocytes, and niche cell interactions to show that hepatic stellate cells, hepatocytes, and sinusoidal endothelial cells collectively instruct Kupffer cells differentiation, implicating BMP, Notch, and other local signals [29]. Spatial proteogenomics further resolved conserved hepatic macrophage niches and demonstrated that anatomical position adds information unavailable from a dissociated cell transcriptional label alone [32]. Together, these studies establish three experimentally separable explanations for a macrophage state observed after MASH improvement: previously exposed cells may survive; their replacements may acquire a similar state; or an incompletely restored niche may continue to instruct that state. Functional assays across disease phases are needed to connect lineage, regulatory, and spatial measurements to retained responsiveness. The principal cellular compartments in which exposure-dependent functional changes may arise are summarized in Figure 3.

Surviving Kupffer cells are plausible sites of exposure-dependent regulatory change, but active MASH also destabilizes the population in which such changes would have to persist. Seidman et al. combined macrophage lineage analysis with RNA sequencing, ATAC sequencing, and transcription factor/chromatin immunoprecipitation measurements in diet-induced NASH. Resident Kupffer cells partially lost their identity program, acquired Trem2 and Cd9 expression, and underwent cell death, while adjacent monocyte-derived macrophages developed convergent transcriptional and epigenomic features [30]. At 20 weeks, LXR chromatin immunoprecipitation detected increased binding at more than 1700 sites and decreased binding at more than 1000 sites. The mechanistic finding was a redistribution of LXR activity: AP-1 and EGR-associated regulatory changes, including increased ATF3 occupancy, redirected a transcriptional regulator normally involved in Kupffer cells identity towards disease-associated enhancers. Conditional LXR loss in the Clec4f-expressing compartment also compromised the resident Kupffer cells population, complicating attribution of subsequent transcriptional differences to reprogramming within otherwise equivalent surviving cells [30]. A complementary depletion and repopulation study by Sakai et al. showed that endothelial DLL4-Notch-RBPJ signaling rapidly activated poised enhancers and induced LXRα, with TGF-β family signals and endogenous LXR ligands contributing to acquisition and maintenance of Kupffer cells identity [75]. This comparison places the MASH epigenome within a broader capacity for niche-directed macrophage differentiation. Chromatin remodeling during injury, therefore, supplies a mechanism through which prior exposure could influence subsequent responses, but its duration cannot be inferred from measurements made while the disease-inducing environment remains present. The relevant candidate is the fate-mapped surviving Kupffer cells, assessed through attenuation and renewed challenge, rather than the aggregate Kupffer cells transcriptional cluster.

Recruited monocytes provide a second route by which an episode of steatohepatitis can durably alter the hepatic macrophage compartment. Tran et al. traced the progressive contribution of Ly6C-positive monocytes to the Kupffer cells pool during NASH as embryonically derived Kupffer cells died and their self-renewal became insufficient. These monocyte-derived Kupffer cells were initially less mature and more inflammatory than embryonically derived Kupffer cellss; importantly, they remained after disease regression and acquired mature Kupffer cells markers [35]. Origin also affected the response to lipid overload: embryonically derived Kupffer cells supported hepatic triglyceride storage more efficiently, whereas monocyte-derived Kupffer cells exacerbated liver injury. Persistence after regression consequently establishes an enduring change in cellular composition, although it does not establish that the inflammatory phenotype measured during active disease was retained unchanged. Remmerie et al. resolved another recruited trajectory using CITE-seq, fate mapping, flow cytometry, and microscopy after 12, 24, and 36 weeks of Western diet feeding: monocytes generated both Kupffer cell-like cells and CLEC4F-negative, osteopontin-expressing lipid-associated macrophages (LAMs) in regions with Kupffer cells depletion and increased desmin staining [31]. Daemen et al. strengthened the distinction between identity and composition by pulse-labeling CD115-expressing cells before dietary injury and separately tracing recruited macrophages. After 20 weeks of high-fat feeding, Ccr2 deficiency reduced the CCR2/CX3CR1-associated LAM compartment and hepatic crown-like structures, yet it increased fibrosis despite comparable weight gain and hepatic triglyceride content [3]. This perturbation implicates recruitment-dependent tissue organization and argues against treating every recruited macrophage as fibrogenic. Because CCR2 loss changes monocyte availability and population assembly, however, the experiment cannot isolate an altered response program within the same macrophages.

Recruitment-directed intervention supplies a complementary causal test. Krenkel et al. studied human NASH tissue and experimental progression and regression models using the CCR2/CCR5 antagonist cenicriviroc. Treatment reduced hepatic Ly6C-positive monocyte-derived macrophage infiltration and improved fibrotic steatohepatitis, without directly changing macrophage polarization in the assays examined or delaying fibrosis resolution after injury cessation [76]. These results support a contribution from population recruitment, but a reduction in disease severity after blocking cell entry cannot establish erasure of a retained program within surviving macrophages.

Lung studies illustrate why resident cell memory and persistent changes in population composition require different evidence. Following respiratory viral exposure, Yao et al. identified memory alveolar macrophages with increased glycolytic activity and enhanced chemokine responses on restimulation; effector CD8 T-cell-derived IFN-γ was required for priming but not maintenance, and formation did not depend on monocyte or marrow progenitor replacement [77]. Conversely, Aegerter et al. found that one month after influenza clearance, recruited monocyte-derived alveolar macrophages enhanced IL-6 production and protection against pneumococcal challenge, while exposed resident macrophages remained largely similar to naive cells [78]. The recruited cells persisted but later lost their protective profile. These contrasting designs demonstrate that cell persistence, cellular origin, and persistence of an altered function are separable variables; their hepatic counterparts require direct testing.

### 4.2. Ontogeny-, Location-, and Stage-Dependent Functions of TREM2-Expressing Hepatic Macrophages

LAM, scar-associated macrophages (SAMs), and GPNMB-positive macrophage designations describe overlapping states rather than a single lineage or fixed functional phenotype. Shared lipid-associated features can occur in recruited hepatic macrophages and resident Kupffer cells adopting a LAM-like state, making cellular origin, anatomical location, and disease stage necessary dimensions of functional comparison [31,42]. The original adipose tissue LAM study showed that Trem2 deletion impaired the LAM program and worsened adipocyte hypertrophy, hypercholesterolemia, and glucose intolerance, establishing a compensatory lipid-handling function outside the liver [79]. In human cirrhosis, Ramachandran et al. instead localized TREM2-positive, CD9-positive SAMs to fibrotic regions and cultured sorted primary SAMs for 24 h to collect conditioned medium, which increased fibrillar collagen expression and proliferation in primary human hepatic stellate cells [80]. Fabre et al. provided a more discriminating functional comparison: GM-CSF, IL-17A, and TGF-β1 differentiated human monocytes into macrophages expressing CD9, TREM2, SPP1, GPNMB, and related scar-associated markers. In these non-MASH-specific fibrosis experiments, the differentiated cells degraded collagen IV but not collagen I and enhanced TGF-β1-induced collagen I deposition by activated mesenchymal cells; cytokine blockade reduced macrophage expansion and fibrosis in murine liver or lung injury models [81]. Neither shared markers nor generic matrix remodeling activity, therefore, predicts net scar removal.

Recent human MASLD studies extend this heterogeneity. Li et al. identified a LAM-associated MITF regulon and then used MITF overexpression and 24-h siRNA knockdown in THP-1 cells to alter lipid-handling genes. Overexpression increased oxygen consumption and HGF production, while conditioned medium enhanced HepG2 proliferation and reduced apoptosis [82]. The transfer experiment supports a protective secretory effect, although HGF abundance and receptor–ligand inference alone do not establish that HGF mediates every conditioned medium effect. Boesch et al. identified a different functional combination: IL32-conditioned THP-1 cells increased lipid uptake during 24-h loading with 200 μM palmitate and 400 μM oleate, showed increased endolysosomal activity, and augmented palmitate-induced IL1B while reducing IL10 expression [83]. These experiments identify lipid processing, inflammatory competence, and trophic support as separable outputs, providing a more informative basis for comparing candidate carrier populations than equating their marker-defined states.

Human macrophage subtyping also requires separating resident identity from the functional state superimposed on it. In the cirrhosis atlas of Ramachandran et al., CD163/MARCO-associated Kupffer cell clusters differed in TIMD4 expression: MARCO-positive, TIMD4-negative cells were selectively reduced, whereas TIMD4-positive cell numbers were comparatively preserved [80]. TREM2-positive, CD9-positive, and MNDA-positive scar-associated macrophages instead accumulated within collagen-rich septa and expressed mediators including SPP1, LGALS3, PDGFB, and TNFSF12. Their inferred derivation from circulating monocytes was supported by transcriptional trajectories and RNA velocity, not by experimental human lineage tracing. This evidential distinction matters when comparing them with fate-mapped mouse monocyte-derived Kupffer cells or CLEC4F-negative LAMs [31,35]. Shared TREM2/CD9 expression identifies an overlapping response to tissue injury but cannot establish common ontogeny, equivalent receptor activity, or the same matrix remodeling function. A credible test of retained responsiveness should, therefore, compare populations defined by resident markers, recruitment history, and spatial localization, rather than pooling all TREM2-positive cells or assigning them to an undifferentiated M1/M2 category. Human cirrhosis supplies a mechanistically informative comparator, but its advanced and etiologically heterogeneous lesions cannot be treated as a longitudinal model of MASH remission.

An early disease-specific map also demonstrates the reversibility of marker-defined states. Xiong et al. used single-cell transcriptomics and secretome analysis in healthy and diet-induced NASH mouse livers, identifying TREM2-high NASH-associated macrophages in mouse and human disease. Their abundance was associated with disease severity and changed with pharmacological and dietary interventions [84]. This treatment responsiveness strengthens the link between the state and its disease environment, while providing no isolated cell rechallenge evidence that the remaining TREM2-positive cells carry a retained inflammatory response.

The function of these populations changes with disease stage, making regression experiments essential for interpreting their persistence. Hendrikx et al. used spatial analysis and bone marrow transplantation in complementary dietary models to show that hematopoietic Trem2 deficiency impaired lipid handling and extracellular matrix remodeling, worsening hepatocyte death, steatohepatitis, and fibrosis [41]. Ganguly et al. explicitly examined regression: Alms1-mutant Foz/Foz mice received a Western diet for 12 weeks before switching to chow for an additional 8–12 weeks. LAMs became the dominant macrophage state during regression, whereas the monocyte-derived Kupffer cells compartment contracted. Trem2-deficient mice retained more fibrosis, and isolated hepatic macrophages tested four weeks after diet withdrawal showed reduced degradation of fluorescent DQ type I collagen; wild-type macrophages increased this activity during regression [4]. This functional readout links a disease-associated state to scar clearance rather than relying on collagenase transcripts. However, global Trem2 deletion affects both population development and receptor-dependent function, so the in vivo phenotype cannot be assigned exclusively to LAMs. Population-targeted experiments by De Ponti et al. further assigned dying cell clearance and tissue repair to both recruited LAMs and resident Kupffer cells adopting a LAM-like phenotype [42]. After vertical sleeve gastrectomy, Fredrickson et al. observed increased macrophage lysosomal and efferocytic activity; pair-fed sham controls and Trem2-deficient mice supported a reparative contribution beyond weight loss alone [69].

Yet receptor function can diverge from its transcript abundance. Wang et al. showed that prolonged hypernutrition induced TNF/IL-1β-dependent ADAM17 cleavage of TREM2, impairing clearance of lipid-laden apoptotic hepatocytes [85]. In a subsequent intervention, Dong et al. delivered cleavage-resistant TREM2 mRNA to hepatic macrophages after eight weeks of high-fat, methionine/choline-deficient feeding, with twice-weekly treatment for four weeks, reducing apoptotic cell burden and liver inflammation [86]. Delivery reached both resident and recruited macrophages, and the nutrient-deficient model limits extrapolation to metabolically typical human MASH. Conversely, myeloid Mertk targeting reduced NASH fibrosis, whereas cleavage-resistant MerTK increased it through an ERK–TGF-β1 pathway [87].

Together, these studies distinguish the net activity of a marker-defined population from the causal function of an individual receptor. The profibrogenic effect of conditioned medium from TREM2-positive human SAMs does not establish that TREM2 signaling drives that effect [80]. Conversely, receptor perturbation supports protective TREM2-dependent functions, although constitutive deficiency can alter population development as well as cellular activity [4,41]. Both recruited LAMs and resident LAM-like Kupffer cells can support repair, arguing against a fixed assignment of pathogenic or protective function by origin [42]. Interpretation should, therefore, integrate origin, location, and disease stage with measured inflammatory, efferocytosis, and matrix remodeling outputs. Persistence of a TREM2-positive population during regression does not by itself establish pathogenic training or justify indiscriminate suppression.

### 4.3. Human Cellular Atlases and the Marrow-to-Liver Axis

Human atlases identify clinically relevant candidate populations, but their sampling structure determines which temporal conclusions are justified. MacParland et al. profiled 8444 cells from five human livers and resolved distinct intrahepatic monocyte/macrophage populations, establishing that heterogeneity precedes the imposition of MASH-associated labels [88]. The Li atlas included 61 individuals—10 controls, 17 with metabolic dysfunction-associated steatotic liver and 34 with MASH—but its 540,216 single-cell transcriptomes came from 29 specimens, not all 61; spatial transcriptomics and metabolomics sampled partly overlapping subsets [82]. Boesch et al. generated an initial single-nucleus dataset of 176,150 nuclei from 18 individuals spanning lean and obese normal liver, steatosis, and MASH. In the single-nucleus analysis, GPNMB-positive cells increased from 5.7% of myeloid cells in non-MASH samples to 12% in MASH, while GPNMB-defined subsets differed in Kupffer cells-associated, antigen presentation, and lipid metabolism markers [83]. These are measurements across patients and disease stages, not observations that the same cells survived a treatment response. Technology adds another source of variation: Andrews et al. compared matched single-cell and single-nucleus profiles across 73,295 liver cells/nuclei, finding complementary recovery of parenchymal and immune populations and demonstrating that some lymphocyte identities were resolved only by the single-cell approach [89]. Consequently, between-atlas differences require consideration of tissue acquisition, dissociation, nuclear sampling, and cell annotation alongside biology.

To test whether hepatic myeloid responsiveness persists after metabolic improvement, paired pretreatment and post-improvement biopsies should be linked to functional assays in prospectively collected samples. Histological assessment should record steatosis, ballooning, lobular inflammation, and fibrosis separately, alongside weight trajectory, glycemic control, and treatment exposure. Where clinically and ethically feasible, fresh tissue would be allocated to viable cell isolation, with adjacent tissue retained for histological and spatial assessment. Comparisons should resolve phenotypically defined resident-like and recruited-like macrophage populations rather than pool all hepatic macrophages. These annotations should not be equated with experimentally established ontogeny: in human cirrhosis, the proposed monocyte origin of scar-associated macrophages was supported by transcriptional trajectories rather than direct lineage tracing [80].

Isolated populations would then be compared under common culture conditions, using a recovery interval validated for viability, basal activation, and identity retention. Environmental regulation of macrophage enhancer programs provides a reason to evaluate these properties rather than assume that culture preserves the original tissue state [33,34]. Basal cytokine secretion and the increment induced by a standardized secondary challenge should be measured separately and normalized to viable cell numbers. An accompanying efferocytosis or lipid-processing assay would help determine whether altered inflammatory responsiveness coexists with preserved repair functions. Retention of an altered response after removal from the diseased tissue would support a persistent cellular phenotype, but residual intracellular cargo and treatment carryover would remain alternative explanations. Loss of the phenotype in culture would likewise be inconclusive because environmental dependence and culture-associated loss of cellular programming could produce similar results. Linking the retained response to subsequent clinical recurrence would require longitudinal follow-up.

Finally, an altered hepatic macrophage response could be continuously supplied from hematopoietic progenitors rather than retained exclusively in mature liver cells. This possibility has experimental support in other systems that is stronger than a simple association between metabolic disease and increased myelopoiesis. Kaufmann et al. showed that BCG exposure reprogrammed bone marrow hematopoietic stem cells and multipotent progenitors; transplantation, parabiosis, and adoptive transfer approaches linked this program to macrophage progeny with improved control of virulent Mycobacterium tuberculosis [37]. Mitroulis et al. found that β-glucan administration expanded myeloid progenitors with IL-1β/GM-CSF-associated signaling and metabolic adaptations, improving responses to secondary LPS challenge and recovery from chemotherapy-induced myelosuppression [36]. Human evidence provides a complementary time scale: Cirovic et al. compared 15 BCG-vaccinated volunteers with five placebo recipients, sampled bone marrow before vaccination and at day 90, and linked progenitor transcriptional changes to peripheral monocyte epigenetic remodeling and enhanced IL-1β release by restimulated peripheral blood mononuclear cells [90]. Metabolic models are particularly relevant to MASH. In Ldlr-deficient mice, four weeks of a Western diet followed by four weeks of chow allowed serum inflammation to subside while augmented myeloid responses and progenitor reprogramming persisted; Nlrp3 deletion prevented the diet-induced program [15]. Edgar et al. cultured macrophages from diabetic donors in physiological glucose and transplanted diabetic bone marrow into normoglycaemic Ldlr-deficient recipients, where aortic root atherosclerosis increased [16]. Culture and transplantation separated previous glycemic exposure from the current systemic environment, although whole-marrow transfer did not isolate a single progenitor subset. These findings justify testing a marrow-to-liver route in MASH, with lineage-resolved progeny and hepatic functional endpoints. Whether the recovering liver maintains those programs also depends on its stromal and extracellular matrix environment.

## 5. Hepatic Injury and Fibrotic Niches Sustaining Myeloid Dysfunction Following Metabolic Improvement

### 5.1. Residual Injury, Efferocytosis, and Stellate Cell Feedback

Metabolic improvement does not necessarily restore the hepatic environment on the same timescale as it reduces steatohepatitis. This distinction is apparent in paired biopsy studies, although persistent fibrosis must not be equated with persistent inflammation. Pais et al. studied 196 patients undergoing bariatric surgery for advanced NASH, of whom 66 underwent repeat biopsy after 6 ± 3 years. Among patients with baseline advanced fibrosis, advanced fibrosis persisted in 47% despite NASH resolution and some histological regression; these patients also had less weight loss and lower rates of metabolic comorbidity remission [91]. The study, therefore, documents incomplete structural recovery, rather than proving inflammation autonomous from all metabolic abnormalities. In a prospective cohort of 180 patients, Lassailly et al. obtained five-year biopsies from 64 of 94 patients who had reached that follow-up point. NASH resolved without worsening fibrosis in 84%, whereas fibrosis decreased in 70.2% and disappeared in 56%; fibrosis improvement continued between years one and five [6]. Baseline severity, follow-up timing, and biopsy availability constrain comparisons between the cohorts. Nevertheless, the separation of activity and fibrosis responses identifies a clinically relevant interval in which scar architecture and resident stromal populations may remain abnormal after substantial disease improvement. The question is whether these residual features continue to instruct myeloid function, impede repair, or alter responses to renewed injury. None of these possibilities can be inferred from a fibrosis score alone; they require functional measurements linked to the recovering tissue environment.

Independent biopsy studies show that distinct histological components also respond differently to interventions beyond bariatric surgery. In the 52-week lifestyle study by Vilar-Gomez et al., 293 patients entered the intervention, and 261 had paired biopsies; across the enrolled cohort, 25% achieved NASH resolution, 47% improved their activity score, and 19% showed fibrosis regression [92]. In the 24-week ENLIVEN trial in biopsy-confirmed NASH with F2-F3 fibrosis, pegozafermin at 30 mg weekly or 44 mg every two weeks produced fibrosis improvement without worsening NASH in 26% and 27%, respectively, versus 7% with placebo; NASH resolution without worsening fibrosis occurred in 23% and 26% versus 2% [93]. These studies establish that inflammatory activity and scar regression are distinct response measures. They cannot determine whether nonresponse reflects continuing metabolic injury, a persistent niche, or trained myeloid function.

NAD^+^-directed interventions additionally illustrate why the cellular target must be specified before attributing disease improvement to myeloid resetting. Liu et al. inhibited α-amino-β-carboxymuconate-ε-semialdehyde decarboxylase (ACMSD), a liver- and kidney-enriched enzyme that diverts intermediates away from tryptophan-derived de novo NAD^+^ synthesis [94]. The inhibitor TLC-065 increased NAD^+^ and mitochondrial respiration in hepatocyte systems; therapeutic administration at 25 mg/kg/day in Western diet-fed, thermoneutrally housed mice reduced hepatic inflammation, fibrosis, and DNA damage signals. Experiments in primary hepatocytes and human liver organoids supported a direct hepatocellular component. In palmitate-stressed organoids, TLC-065 reduced nuclear phosphorylated H2A.X and 53BP1 foci, while treated mouse livers showed lower excessive poly-ADP ribosylation associated with genomic stress. These results connect NAD^+^ availability to hepatocyte bioenergetics and genome protection rather than identifying a macrophage-specific memory mechanism. Compared with the PHGDH experiments, in which NAD^+^ accumulation accompanied deacetylase-dependent restriction of macrophage IL-1β, the ACMSD intervention acts on a different cellular and biochemical context [95]. Reduced hepatocyte injury could diminish the signals sustaining hepatic myeloid activation, but lower inflammation after treatment cannot distinguish this indirect route from altered intrinsic myeloid responsiveness. Evidence for memory resetting would require a post-treatment, stimulus-free assessment of defined macrophage populations followed by rechallenge.

In addition to the production of injured cells, their removal determines the local burden of potentially inflammatory material. When apoptotic hepatocytes are inefficiently cleared, reducing lipid input may leave a local source of inflammatory material whose duration depends on clearance capacity. Wang et al. demonstrated that hepatocyte-derived sphingosine-1-phosphate induced macrophage TREM2 and supported efferocytosis of lipid-laden apoptotic hepatocytes. With prolonged hypernutrition, however, hepatic TNF and IL-1β promoted ADAM17-dependent receptor cleavage, compromising clearance and increasing the accumulation of dying hepatocytes [85]. This mechanism connects an extracellular inflammatory environment to loss of a macrophage disposal function and renewed cytokine production, without requiring a stable program retained after exposure removal. Dong et al. subsequently tested whether restoring that disposal function could interrupt disease. Their synthetic cleavage-resistant receptor joined the TREM2 ligand-binding domain to DAP12 signaling elements and was delivered as mRNA in liver-targeted lipid nanoparticles. After eight weeks of high-fat, methionine/choline-deficient feeding, twice-weekly administration for four weeks reduced apoptotic cell burden and inflammatory liver pathology [86]. Delivery reached resident and recruited macrophages, so the improvement cannot be assigned to one lineage; the nutrient-deficient model also differs from metabolically typical human MASH. Together, these experiments identify defective injury clearance as an actionable source of ongoing local stimulation. They do not establish how long apoptotic material remains after successful metabolic treatment because the relevant intervention experiments were performed in induced disease rather than after a demonstrated stimulus-free recovery interval. Residual cell death, extracellular debris, and functional efferocytosis, therefore, need to be measured separately during clinical or experimental remission.

Activated hepatic stellate cells (HSCs) can change both the matrix surrounding macrophages and the functions those macrophages acquire, but the direction of this instruction is not uniformly pathogenic. Chan et al. isolated HSCs from mice after four months of MASH-inducing diet and cultured them above naive bone marrow-derived macrophages in 0.4 μm pore transwells for five days. After removing the inserts and replacing the medium, macrophages showed increased Trem2, Gpnmb, Cd63, and Spp1 expression and greater degradation of fluorescent DQ collagen, detectable within 30 min of substrate addition [96]. Thus, an injury-associated stromal population could induce a LAM-like, collagen-processing phenotype in previously unexposed macrophages without direct cell contact. The medium-change experiment supports a short-term acquired functional state, although the unidentified soluble mediators and absence of prolonged withdrawal prevent conclusions about its persistence. A contrasting cytokine environment was reconstructed by Fabre et al.: GM-CSF, IL-17A, and TGF-β1 differentiated human monocytes into scar-associated macrophages that degraded collagen IV but not collagen I and increased TGF-β1-driven collagen I deposition by mesenchymal cells. Blocking these cytokines reduced macrophage expansion and fibrosis in murine hepatic or pulmonary injury [81]. The collagen substrate and mesenchymal response, therefore, determine whether apparently similar scar-associated programs support removal or production of matrix. Stromal intervention provides a further separation of outcomes. Kim et al. profiled 18 human livers using single-nucleus RNA and chromatin accessibility assays and then knocked down SERPINE1 or SPON1 specifically in HSCs before incorporating them into metabolically challenged human liver spheroids. Both interventions reduced COL1A1 and ACTA2; PAI-1 inhibition with TM5441 at 20 or 40 μM also suppressed collagen production [97]. Their Lrat-Cre-directed Serpine1 deletion was tested in CCl4-induced fibrosis, where scarring decreased without a corresponding reduction in the reported inflammation measures. This supports HSC-directed control of fibrogenesis, while leaving macrophage recovery and the effect of HSC-specific Serpine1 loss in metabolic injury unresolved.

Mechanistic fibrosis experiments distinguish stromal sensitization from survival of already activated cells. In Tlr4-chimeric mice and LPS-challenged HSCs, Seki et al. localized a profibrogenic response to stellate cell TLR4: MyD88-NF-κB signaling reduced the TGF-β pseudoreceptor BAMBI and increased responsiveness to TGF-β [98]. Pradere et al. instead showed that macrophage-derived IL-1 and TNF activated NF-κB in HSCs and supported the survival of activated myofibroblasts. Cytokine neutralization in coculture and receptor ablation in vivo strengthened this causal assignment; the cytokines did not directly induce HSC activation in those assays [99]. Thus, macrophage–HSC interactions can prolong a fibrogenic niche through distinct mechanisms. These non-MASH fibrosis experiments support biological plausibility without establishing their duration after metabolic remission.

### 5.2. Matrix Mechanics, Spatial Organization, and Resolution

The physical matrix is another instructive variable, but its effects must be resolved by cell type and function. Georges et al. measured fresh-liver shear storage modulus during twice-weekly CCl4 injury in rats, sampling from days 3 to 70. Stiffness rose before readily detectable matrix accumulation, and lysyl-oxidase inhibition with β-aminopropionitrile partially limited the early increase [100]. Matrix quantity, therefore, did not fully explain tissue mechanics. In primary HSC cultures, Olsen et al. varied polyacrylamide support stiffness and matrix coating: compliant supports maintained a less activated phenotype, whereas approximately 12 kPa supports promoted spreading and α-smooth muscle actin organization, with matrix adhesion also required [101]. These are stromal results and cannot be transferred directly to macrophages. Atcha et al. tested macrophages on fibronectin-conjugated substrates spanning 1, 20, 40, and 280 kPa, combined with defined IFNγ/LPS or IL-4/IL-13 stimulation. Piezo1-dependent calcium signaling modulated activation, and myeloid Piezo1 deletion reduced IFNγ/LPS-induced TNF and IL-6 secretion [102]. The extreme stiffness range and soluble agonists delimit its relevance to a recovering liver. By contrast, Wang et al. examined apoptotic cell uptake across 0.2–64 kPa substrates and found that increasing stiffness enhanced macrophage efferocytosis in a Piezo1-dependent manner. After eight CCl4 injections, myeloid Piezo1 deletion produced comparable fibrosis at 24 h after the final dose but greater fibrosis at 48 h, alongside defective apoptotic cell clearance [103]. This short post-injury window supports impaired early recovery, not long-term relapse susceptibility; Lyz2-directed deletion also extends beyond macrophages. The studies are consequently compatible with mechanical cues amplifying inflammatory responses under one stimulus and supporting disposal of dying cells under another. Tissue stiffness alone is insufficient to predict either outcome. Adhesive ligand presentation, cargo identity, soluble signals, and the timing of measurement must accompany any claim that residual matrix maintains myeloid dysfunction.

Spatial organization determines which of these inputs a macrophage encounters and which cells receive its products. Itoh et al. localized hepatic crown-like structures around injured lipid-laden hepatocytes in Mc4r-deficient mice and human NASH, linking these aggregates to surrounding fibrogenesis [68]. Ioannou et al. fed mice a 15% fat diet containing 0–1% cholesterol for six months; cholesterol crystals, crown-like structures, and fibrosing steatohepatitis emerged at dietary cholesterol concentrations of at least 0.5%. In a complementary coculture, LDL-loaded HepG2 cells developed lipid droplet cholesterol crystals and increased TNF, NLRP3, and IL1B expression and IL-1β secretion in THP1 macrophages [67]. These observations define focal injury environments but do not show that the structures survive metabolic remission. Their functional meaning is constrained by Daemen et al.’s recruitment experiment: after 20 weeks of high-fat feeding, Ccr2 deficiency reduced crown-like structures and the associated recruited macrophage compartment while increasing fibrosis despite comparable hepatic triglyceride content [3]. A lesion-associated aggregate may, therefore, contain an adaptive response to damage. Guilliams et al. also identified LAMs near bile ducts in the healthy liver and their expansion into steatotic regions during obesity, demonstrating that the same broad program occupies distinct anatomical environments [32]. Within human cirrhotic scars, Ramachandran et al. localized TREM2-positive/CD9-positive macrophages, specialized endothelial cells, and PDGFRα-positive mesenchymal cells. Recombinant TNFSF12 and PDGF-BB increased primary human HSC proliferation, which was reduced by cognate receptor blockade; endothelial–HSC coculture experiments additionally supported Notch-dependent collagen production [80]. These perturbations strengthen selected communication pathways beyond spatial colocalization, although they do not establish every predicted interaction or its persistence after treatment. In human MASLD, Li et al. integrated spatial transcriptomics with metabolomics in adjacent tissue sections, associating fibrotic regions with altered endothelial–HSC programs and local phospholipid accumulation [82]. Such maps identify candidate exposure sites; they do not directly measure the extracellular ligands accessible to individual macrophages. The relevant spatial hypothesis is, therefore, that residual lesion architecture concentrates particular interactions, rather than that proximity or shared markers demonstrate an autonomous inflammatory program.

Endothelial remodeling provides an additional mechanism by which a residual scar could maintain myeloid exposure without autonomous macrophage memory. Ramachandran et al. identified expansion of CD34-positive, PLVAP-positive endothelial populations expressing either VWA1 or the atypical chemokine receptor ACKR1, alongside loss of the CD34-negative, CLEC4M-positive sinusoidal endothelial population [80]. Under flow, endothelial cells from cirrhotic liver supported increased leukocyte transmigration; ACKR1 knockdown attenuated this response. This functional perturbation links an endothelial receptor to cell entry more directly than endothelial–macrophage proximity or predicted ligand–receptor interactions. Its implication differs from the DLL4–Notch–LXRα pathway that instructs Kupffer cell identity: one mechanism changes access to the hepatic compartment, whereas the other changes the differentiation program of cells occupying it [75]. Persistent vascular remodeling could consequently sustain recruitment into a resolving lesion and alter the apparent macrophage response at the population level. The cirrhosis experiments, however, did not establish persistence of ACKR1-dependent recruitment after metabolic improvement or demonstrate that recruited cells acquire a stimulus-independent memory state.

Recovery experiments show that dismantling a fibrotic environment requires active cellular functions and that its disappearance need not restore every participating cell to its original state. Duffield et al. used CD11b-DTR-mediated macrophage depletion in a reversible CCl4 model: depletion during established injury reduced scarring and myofibroblasts, whereas depletion during recovery impaired matrix degradation [104]. Ramachandran et al. subsequently combined adoptive transfer, in situ labeling, and depletion to identify recruited Ly6C-low restorative macrophages; cellular debris uptake reproduced parts of this program in vitro, and liposome administration during recovery increased restorative macrophages and accelerated scar removal [105]. These chemical injury studies establish stage-dependent function. Ganguly et al. extended the argument to dietary MASH regression in Foz/Foz mice: after Western diet withdrawal, macrophage collagenolytic activity increased, whereas Trem2 deficiency left greater residual scar despite similar downregulation of collagen gene expression [4]. Existing matrix removal was, therefore, distinguishable from reduced matrix synthesis. A recent study by Li et al. identified an additional endothelial–stromal contribution. After six weeks of combined Western diet, sugar water, and weekly CCl4—the FAT-MASH protocol—all inducing components were withdrawn for two or six weeks. Recombinant SFRP2 given twice weekly delayed spontaneous fibrosis regression at two weeks, but this difference disappeared by six weeks; activity-based probes localized serine protease activity to scar-associated HSCs and endothelial cells [106]. Systemic SFRP2 administration did not establish cell-specific Wnt9b causality, and toxin supplementation constrains extrapolation to dietary disease alone. Meanwhile, lineage studies demonstrate incomplete stromal restoration. Kisseleva et al. genetically labeled activated HSCs and showed that a substantial fraction survived regression, downregulated fibrogenic genes, and reactivated more readily on renewed stimulation [107]. Troeger et al. used Vimentin-CreER tracking and single-cell PCR to document HSC deactivation; cells isolated 45 days after the last CCl4 injection responded more strongly than naive HSCs to 48-h TGF-β or PDGF stimulation, despite near-normal baseline activation markers [108]. This is evidence of retained stromal responsiveness, not trained myeloid immunity. A recovering liver may consequently contain effective repair macrophages alongside stromal cells with altered subsequent responsiveness; neither scar reduction nor low basal inflammation establishes a complete biological reset.

The extracellular component of recovery was resolved experimentally by Iredale et al. in rats after four weeks of twice-weekly CCl4 exposure. Sampling at 0, 3, 7, and 28 days after withdrawal showed scar remodeling toward control levels, a twelvefold reduction in activated HSCs, and evidence of HSC apoptosis. TIMP-1 and TIMP-2 expression declined while collagenase activity increased [109]. This time course links removal of matrix-producing cells and reduced protease inhibition to scar degradation. It provides a recovery benchmark for the niche, while leaving myeloid responsiveness after renewed metabolic injury unmeasured.

These findings favor a model in which tissue instruction, changes in cellular composition, and cell-autonomous adaptation can coexist, but each requires a different experimental test. Niche availability itself changes the population under study: Scott et al. depleted Kupffer cells with diphtheria toxin and demonstrated durable engraftment and self-renewal of recruited monocytes, while Bonnardel et al. used depletion/repopulation experiments to identify endothelial, hepatocyte and HSC signals that impose Kupffer cells identity [29,74]. These homeostatic experiments establish the capacity of a liver environment to instruct incoming cells; they do not demonstrate that a post-MASH niche imposes an inflammatory phenotype. Chan’s transwell experiment provides the more disease-relevant comparison by exposing naive macrophages to MASH-derived HSCs, but its brief medium-replacement step still leaves long-term retention unresolved [96]. A decisive recovery study would, therefore, compare fate-mapped surviving cells, newly recruited cells, and naive cells exposed to the recovering niche. Function should be reassessed after controlled withdrawal from that environment, with residual lipid, cytokines, and matrix-associated ligands quantified, and with matched culture conditions that minimize additional activation. Reciprocal transfer or reconstituted coculture can test whether the altered state follows the donor cell or the recipient environment, while timed stromal or matrix interventions can determine whether tissue restoration changes the response to renewed injury. Cell depletion, transplantation, and prolonged plastic culture each disturb identity or niche occupancy, so their effects require appropriate controls. Paired human biopsies can locate residual injury and stromal states, but causal interpretation still depends on functional testing rather than histological persistence alone. Only after these extracellular and compositional contributions are addressed can metabolic and chromatin changes within myeloid cells be evaluated as mechanisms that retain exposure-dependent function.

## 6. Immunometabolic and Epigenetic Mechanisms Underlying Persistent Myeloid Responsiveness

The mechanistic evidence should be evaluated according to both disease relevance and temporal design. Chromatin profiling during experimental NASH identifies regulatory changes in hepatic macrophages but does not establish their functional persistence after regression [30]. Conversely, rested monocyte models demonstrate altered secondary responsiveness after microbial or lipid conditioning, providing experimental precedents for training and tolerance outside the MASH setting [19,24]. These evidence streams are complementary: disease-specific studies identify candidate mechanisms, whereas withdrawal rechallenge designs test whether an altered response outlasts the initiating exposure. The following subsections distinguish these contributions and identify which post-regression measurements remain necessary.

**Figure 4:**
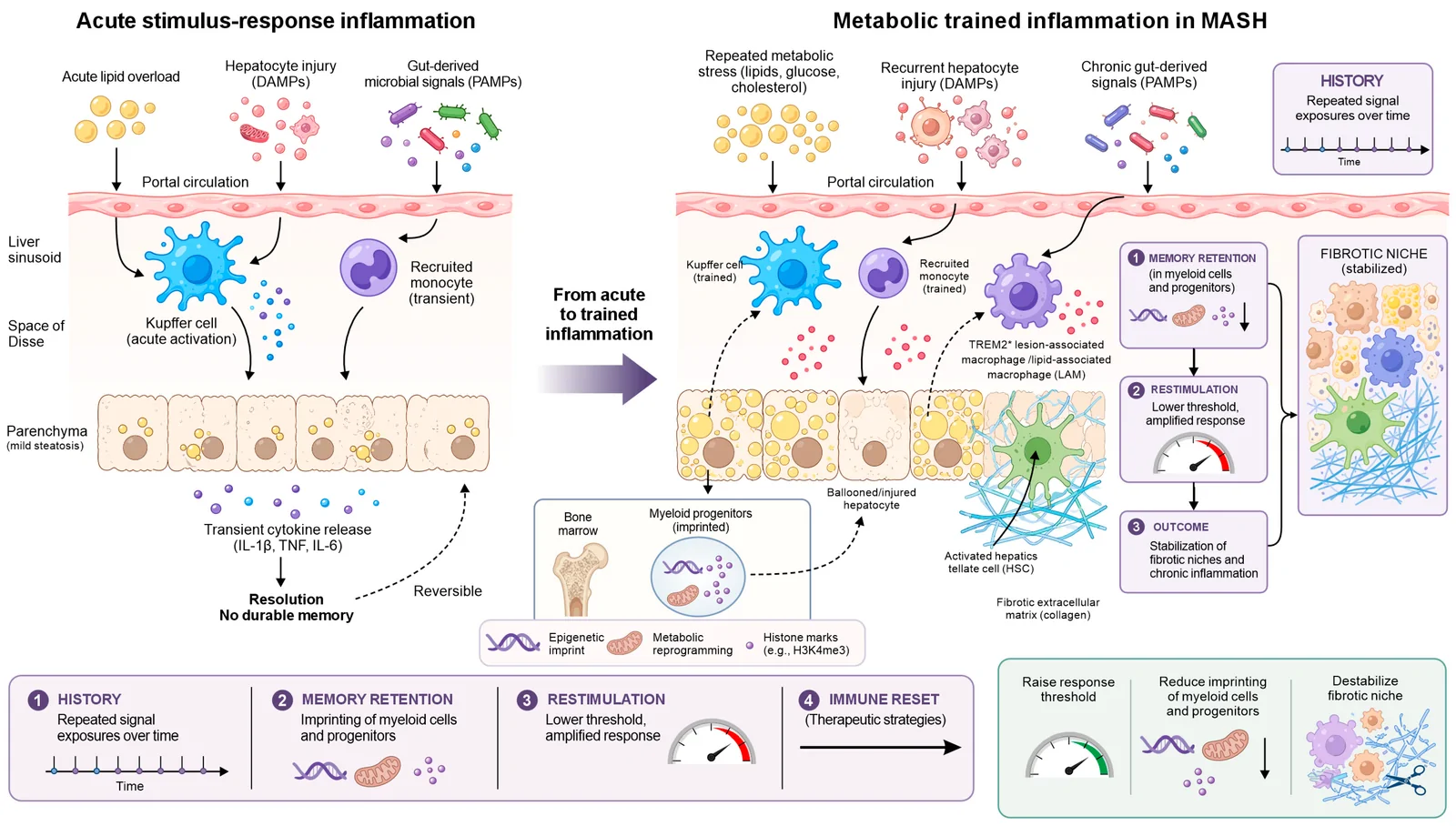
Metabolic and chromatin pathways implicated in myeloid responsiveness. Metabolic pathways influence substrate availability, chromatin-modifying enzymes, and inflammatory transcription. The schematic integrates mechanisms from experimental training models and liver disease studies; their evidential support differs. Histone changes and pathway activation alone do not establish retained responsiveness in MASH. Persistence, altered rechallenge responses, and causal dependence require separate validation. ACLY, ATP citrate lyase; GLS, glutaminase; LDH, lactate dehydrogenase; TCA, tricarboxylic acid.

Novakovic et al. further showed that β-glucan administered after LPS could restore approximately 60% of tolerized genes upon later LPS re-exposure, with recovery of enhancer H3K27ac; a BET inhibitor that prevented tolerance when administered with the initial LPS exposure did not reverse established tolerance [27]. Prevention, reversal, and suppression of secondary cytokine output, therefore, represent different biological effects. In MASH, these distinctions require interventions timed to initial conditioning, maintenance during regression, and secondary challenge. Figure 4 summarizes the metabolic–chromatin connections, whereas Table 2 distinguishes the demonstrated function of each pathway from its unresolved contribution to post-regression responsiveness.

### 6.1. Glycolytic Control of Inflammatory Output

Glycolytic and mevalonate-dependent programs provide experimentally supported mechanisms for establishing trained responsiveness, but their contribution depends on stimulus identity and intervention timing. In β-glucan-trained human monocytes, Cheng et al. observed increased glucose consumption and lactate production, activation of the Akt–mTOR–HIF-1α pathway, and reduced oxygen consumption in the experimental conditions examined. Rapamycin restricted to the initial training period reduced cytokine responses measured several days later, while myeloid HIF-1α deficiency abolished β-glucan-mediated protection against subsequent bacterial infection [12]. These interventions support a requirement during establishment of the response; they do not demonstrate that inhibiting the same pathway reverses an already maintained inflammatory state. Bekkering et al. identified mevalonate, rather than cholesterol itself, as a mediator linking cholesterol biosynthesis to IGF1 receptor–mTOR signaling and epigenetic remodeling. Pharmacological interference with this pathway impaired training, and monocytes from patients with mevalonate kinase deficiency provided a human genetic context associated with enhanced inflammatory and epigenetic responsiveness [23]. However, mitochondrial suppression is not a universal feature. Keating et al. treated human monocytes with 10 μg/mL oxLDL for 24 h, washed them, and assessed them after five days of rest. Both extracellular acidification and oxygen consumption increased before LPS re-exposure; inhibiting glycolysis during the initial exposure reduced subsequent TNF and IL-6 production and associated H3K4me3 enrichment [45]. BCG-trained monocytes likewise increased glycolytic and oxidative metabolism [121]. These comparisons favor stimulus-specific metabolic coordination over a compulsory switch from oxidative phosphorylation to glycolysis. A direct MASH example comes from Lu et al.: conditioned medium from palmitate-treated hepatocytes induced macrophage F13A1-dependent PKM2 nuclear translocation and glycolytic activation. Macrophage-directed F13A1 knockdown initiated at week 6 of a choline-deficient, amino acid-defined high-fat diet reduced hepatic inflammation and fibrosis without reducing hepatic triglyceride accumulation or steatosis [43]. This separation supports a macrophage metabolic contribution to injury beyond bulk lipid deposition. Because the dietary insult continued and no stimulus-free rechallenge was performed, it establishes a mechanism of ongoing MASH inflammation, rather than retained training or its erasure.

A second MASH intervention illustrates that enzyme conformation can matter alongside pathway abundance. In high-fat-diet-fed mice and activated macrophages, Xu et al. reported that annexin A5 interacted with PKM2, reduced its Y105 phosphorylation, and promoted tetramer formation, accompanying a shift from glycolysis toward oxidative phosphorylation and improvement of steatosis, inflammation, and fibrosis. PKM2 inhibition and a Y105E phosphomimetic construct supported dependence on PKM2 activity [110]. These results complement the F13A1–PKM2 pathway by testing a different regulatory intervention, but treatment during active disease establishes metabolic control of inflammation rather than reversal of a rested memory state.

### 6.2. Tricarboxylic Acid Metabolites and Regulatory Enzymes

Tricarboxylic acid cycle metabolites influence inflammatory responsiveness through distinct biochemical routes, and their accumulation cannot be interpreted as a common proinflammatory signature. Arts et al. found increased fumarate and reduced KDM5 histone demethylase activity in β-glucan-trained monocytes. A 24 h exposure to 100 μM monomethyl fumarate, followed by five days without the initiating stimulus, increased cytokine production upon LPS or Pam3Cys stimulation and reproduced part of the training-associated H3K4me3 and H3K27ac pattern. Succinate and malate did not produce the same functional training effect under the conditions tested; α-ketoglutarate counteracted fumarate-associated inhibition of KDM5 and attenuated the enhanced cytokine response [22]. These comparisons connect metabolite identity, an epigenetic enzyme, and a delayed functional phenotype more convincingly than metabolite abundance alone. Succinate has a different evidential profile. Tannahill et al. demonstrated that succinate accumulation in LPS-activated macrophages supported HIF-1α-dependent IL-1β expression, whereas glycolytic inhibition did not suppress TNF in the same manner [111]. Mills et al. subsequently showed that inhibiting succinate dehydrogenase with dimethyl malonate increased succinate abundance while reducing HIF-1α and IL-1β responses; mitochondrial reactive oxygen species generated during succinate oxidation contributed to the inflammatory effect [112]. Thus, oxidation and electron transport can be more informative than the metabolite pool itself. Conversely, Domínguez-Andrés et al. linked the IRG1–itaconate pathway to innate tolerance and showed that β-glucan could counteract this program [113]. Liu et al. demonstrated that glutamine-derived α-ketoglutarate supported IL-4-associated macrophage polarization through JMJD3, but its effects on LPS tolerance involved a distinct, JMJD3-independent mechanism regulating inflammatory signaling [122]. These findings should not be collapsed into a single demethylation model. For MASH, fumarate–KDM5 coupling is a plausible mechanism for a retained inflammatory response, whereas succinate–mitochondrial ROS and itaconate-dependent suppression may regulate contemporaneous activation or tolerance. Establishing which process persists after hepatic metabolic improvement requires functional temporal evidence, rather than assigning memory to every altered metabolite.

Independent biochemical evidence helps explain the anti-inflammatory direction of the itaconate pathway. Mills et al. showed in mouse and human macrophage systems that itaconate alkylated KEAP1 cysteines and enabled NRF2-dependent antioxidant and anti-inflammatory gene expression; the cell-permeable derivative 4-octyl itaconate reduced cytokine production and protected against LPS-induced lethality [114]. The derivative-based intervention supplies a functional test, while its pharmacology and the contemporaneous LPS setting limit extrapolation to endogenous itaconate-mediated erasure of MASH-associated memory.

### 6.3. Lipid Processing and Functionally Divergent Macrophage Responses

Lipid handling can enhance or restrain macrophage responsiveness, depending on lipid species, delivery route, and intracellular processing. Seufert et al. pre-exposed bone marrow-derived macrophages to albumin-bound palmitate for 12–24 h, removed the extracellular fatty acid, and then applied LPS. Prior palmitate exposure enhanced TNF, IL-6, and IL-1β secretion; co-treatment with the ceramide synthesis inhibitor fumonisin B1 attenuated these responses [73]. This supports ceramide-dependent sensitization, but the immediate transition to LPS stimulation, without a multiday recovery interval, leaves stronger uncertainty about durable training than the rested oxLDL experiments. It also cautions against treating secreted cytokine abundance and transcript induction as interchangeable endpoints. Zhou et al. obtained an opposing response using lipid droplets isolated from steatotic livers: macrophages exposed to these droplets for 3 h and subsequently cultured without extracellular droplets displayed reduced inflammatory responses, including diminished TNF/IL-12 production and inflammasome activation. TREM2 was dispensable for droplet uptake but required for the suppressive response, while inhibition of lysosomal acid lipase did not eliminate suppression [115]. Lal deletion reduced lipid droplet hydrolysis and free fatty acid release without abolishing inhibition of NLRP3 activation. Droplet exposure also reduced MS4A7 expression and its physical association with NLRP3; Ms4a7 deletion diminished inflammasome activation, with little further suppression by droplet exposure. By contrast, Trem2 deficiency increased ASC speck formation and weakened droplet-associated suppression of IL-1β cleavage [115]. These genetic and biochemical comparisons distinguish cargo uptake, lysosomal processing, and inflammasome regulation, even within a TREM2-associated cellular state. The short conditioning interval and culture-based suppression assays do not establish tolerance maintained through MASH remission. In complementary bone marrow transplantation models, Hendrikx et al. found that hematopoietic TREM2 deficiency impaired lipid handling and extracellular matrix remodeling and aggravated steatohepatitis, cell death, and fibrosis [41]. Lipid-associated macrophage differentiation can, therefore, serve an adaptive function within a damaged liver; neither TREM2 expression nor intracellular lipid accumulation establishes pathogenic training. Claims concerning fatty acid oxidation require additional pharmacological scrutiny. Nomura et al. showed that macrophage Cpt2 deletion abolished long-chain fatty acid oxidation without preventing IL-4-induced CD301/CD206 expression or induction of Arg1, Mgl2, and Retnla. High-dose etomoxir nevertheless inhibited polarization in both control and Cpt2-deficient cells [123]. Divakaruni et al. linked such high-dose effects to off-target metabolic disruption, including depletion of the free coenzyme A pool [124]. These studies concern alternative activation rather than MASH memory, but they demonstrate why an etomoxir-sensitive phenotype cannot by itself establish a fatty acid oxidation-dependent retained state. A credible lipid memory mechanism must distinguish the initiating lipid signal, persistent intracellular substrate, altered cell differentiation, and a response that remains after those substrates and signals have subsided.

### 6.4. Chromatin Persistence, Acetyl-CoA, Serine, and One-Carbon Metabolism

Chromatin remodeling becomes evidence for memory when its persistence is linked to a delayed response, rather than merely detected in an inflamed tissue. Ostuni et al. identified latent macrophage enhancers that acquired transcription factor occupancy and histone modifications after stimulation, retained selected marks after stimulus withdrawal, and responded more rapidly or strongly to renewed stimulation [25]. Saeed et al. similarly distinguished persistent H3K4me1 from more dynamic H3K27ac during monocyte differentiation, providing a basis for separating a retained regulatory configuration from current enhancer activity [19]. However, a retained mark is still a candidate substrate unless perturbation changes the subsequent functional response. Keating et al. addressed this relationship using SETD7/Set7 inhibition during a 24 h β-glucan exposure, followed by five days of rest. Cyproheptadine reduced later TNF and IL-6 responses and H3K4me1 enrichment at regulatory regions associated with SDHB and MDH2, while Set7-deficient mice supplied orthogonal genetic evidence for impaired training [125]. The convergence is stronger than chromatin association alone, although cyproheptadine has additional pharmacological actions and SETD7 modifies nonhistone substrates; these experiments do not assign the entire phenotype specifically to histone methylation at either locus. In liver disease, Seidman et al. integrated lineage information, transcriptomics, chromatin accessibility, and transcription factor binding during prolonged experimental NASH. Resident Kupffer cells lost aspects of their hepatic identity while AP-1/EGR-associated regulatory changes and altered LXR activity accompanied disease-associated macrophage states [30]. These observations establish extensive niche-dependent regulatory remodeling during ongoing injury. They do not show that the remodeled state persists functionally after removal of the disease environment. The same distinction applies to Boesch et al.’s human liver atlas, which analyzed 176,150 nuclei from 18 lean, obese, MASL, and MASH livers and combined cellular profiles with spatial and proteomic information [83]. Its disease-associated myeloid states identify cells and regulatory programs for subsequent testing, but cross-sectional differences cannot resolve prior exposure, cell replacement, or responses after metabolic improvement.

Acetyl-CoA availability links metabolic activity to chromatin regulation, but the resulting transcriptional program depends on the initiating signal. Covarrubias et al. showed that IL-4 recruits Akt–mTORC1 signaling to regulate ATP citrate lyase (ACLY), supporting histone acetylation and selected alternative activation genes involved in proliferation and chemokine production [116]. Lauterbach et al. used metabolic tracing to show that early TLR4 stimulation redirects glucose-derived carbon towards acetyl-CoA and histone acetylation; MyD88- and TRIF-dependent ACLY activation supported selected LPS-responsive gene programs [117]. ACLY supplies acetyl-CoA through ATP-dependent cleavage of citrate into acetyl-CoA and oxaloacetate. These experiments show that the same metabolic enzyme can support different transcriptional outputs and should not be assigned an exclusively proinflammatory function. They establish metabolic support for stimulus-dependent transcription, rather than persistence of an exposure-dependent response through MASH regression. Testing the latter would require phase-restricted ACLY perturbation followed by a secondary challenge assay without the inhibitor in a defined hepatic macrophage population.

Serine synthesis adds a distinct connection between inflammatory receptor expression, chromatin regulation, and inflammasome processing. Wang et al. found that NF-κB signaling increased phosphoglycerate dehydrogenase (PHGDH), the rate-limiting enzyme diverting the glycolytic intermediate 3-phosphoglycerate into de novo serine synthesis [95]. Pharmacological inhibition and genetic modulation of PHGDH reduced macrophage IL-1β production, accompanied by NAD^+^ accumulation and increased expression and activity of the NAD^+^-dependent deacetylases SIRT1 and SIRT3. Mechanistically, PHGDH supported H3K9/H3K27 acetylation and TLR4 transcription, thereby influencing the capacity to express IL-1β; it also supported acetylation of NLRP3 and ASC and subsequent inflammasome-dependent IL-1β maturation. These are separate regulatory levels: histone modification changes receptor-dependent transcriptional competence, whereas acetylation of inflammasome components changes post-translational processing of the cytokine. Myeloid-specific Phgdh depletion attenuated LPS-induced systemic inflammation, providing in vivo support beyond a metabolite–cytokine correlation [95]. The findings establish a serine synthesis–NAD^+^–deacetylase axis in inflammatory macrophages, but neither the systemic endotoxin model nor receptor upregulation demonstrates persistence through MASH regression. They also caution against interpreting total cellular acetylation as a specific measure of chromatin-based memory.

One-carbon metabolism connects serine availability to a different chromatin substrate: the methyl donor S-adenosylmethionine (SAM). Yu et al. showed that LPS activation coordinated the pentose phosphate pathway, serine synthesis, and one-carbon metabolism in macrophages [118]. Glucose-derived ribose and one-carbon units supplied through glucose and serine metabolism supported de novo ATP synthesis, linking nucleotide production to the methionine cycle and SAM generation. Perturbing pathways that supplied SAM reduced inflammatory outputs, while the SAM:S-adenosylhomocysteine ratio supported H3K36 trimethylation and IL-1β production. This mechanism is biochemically distinct from fumarate-mediated inhibition of KDM5: one changes methyl donor availability, whereas the other inhibits removal of selected histone methylation marks [22]. Likewise, H3K36me3 cannot be substituted for H3K4me1 or H3K4me3 as an interchangeable marker of training. The serine and one-carbon studies together explain how glycolytic carbon can support receptor expression, cytokine maturation, and chromatin methylation through separate routes [95,118]. Their principal limitation for this review is temporal: metabolic dependence during LPS activation identifies machinery capable of sustaining inflammatory output but does not show that a corresponding regulatory state survives stimulus withdrawal in Kupffer cells or monocyte-derived hepatic macrophages.

NAD^+^-associated effects also require cellular attribution. In macrophages, PHGDH inhibition was associated with NAD^+^ accumulation, increased SIRT1/SIRT3 activity, and reduced IL-1β production [95]. By contrast, the ACMSD intervention discussed in Section 5.1 supports NAD^+^-dependent hepatocyte protection [94]. The same metabolite, therefore, participates in different cellular and regulatory contexts. Reduced hepatic inflammation following hepatocyte protection cannot be interpreted as correction of an intrinsic macrophage memory program without subsequent cell-specific functional testing.

### 6.5. Histone Lactylation and the Temporal Requirements for Memory

Histone lactylation offers a particularly instructive example of how a modification can participate in both current activation and retained responsiveness without carrying a fixed inflammatory meaning. Zhang et al. observed delayed histone lactylation, including H3K18la, during LPS/IFN-γ activation of mouse macrophages. Lactylation increased prominently at 16–24 h and was associated with late induction of genes such as Arg1; disrupting lactate production reduced these effects [119]. This work established a metabolic connection to a late transcriptional program within a single activation episode, rather than a response retained across prolonged stimulus withdrawal. Ziogas et al. examined the latter question in BCG-trained human monocytes: a 24 h exposure followed by five days of rest increased TNF and IL-6 production upon subsequent LPS challenge, while H3K18la was enriched predominantly at active distal regulatory regions. Much of the initial transcriptional response subsided during the rest period, whereas part of the lactylation-associated regulatory pattern remained. Inhibition of lactate dehydrogenase or p300/CBP during the initial exposure reduced later cytokine responsiveness, and monocytes sampled 90 days after human BCG vaccination retained increased H3K18la [46]. This supplies temporal and intervention evidence, although the vaccination result concerns a replenished monocyte population rather than survival of the originally exposed cells. Cai et al. independently used β-glucan training with a five-day rest interval and showed that LDHA disruption altered metabolic flux, histone lactylation, and secondary cytokine responses. Their murine monocyte experiments connected H3K27la enrichment at inflammatory promoters with chromatin accessibility, while isotope tracing demonstrated that lactate could contribute carbon to the tricarboxylic acid cycle and alter glucose allocation to other pathways [120]. The difference between promoter-associated H3K27la and enhancer-associated H3K18la argues against treating total histone lactylation as a single mechanistic readout. Moreover, LDHA perturbation changes redox balance and metabolic flux, and p300/CBP inhibition affects acetylation as well as lactylation; neither intervention selectively proves that one histone lactylation site is necessary and sufficient for training. In MASH, Li et al. reported an HK2–H3K18la-positive feedback program in activated hepatic macrophages, with myeloid Hk2 deletion attenuating pathology in an ongoing methionine- and choline-deficient diet model [44]. H3K18la enrichment at glycolytic gene promoters linked lactate production to reinforcement of glycolytic transcription, while myeloid Hk2 deletion or HIF-1α inhibition disrupted the metabolic–inflammatory circuit [44]. This connects a specific glycolytic enzyme, a histone modification, and inflammatory hepatic macrophage function. However, the reported M1-like inflammatory phenotype is an experimental activation descriptor, not a lineage definition equivalent to resident Kupffer cells, recruited LAMs, or human SAMs. The study provides disease-specific evidence for feedback during ongoing injury, but no verified stimulus-free rechallenge evidence establishes retained responsiveness. The rested BCG and β-glucan experiments, therefore, provide stronger temporal evidence for lactate-associated chromatin remodeling in trained immunity.

The evidence considered here establishes several distinct components of hepatic myeloid adaptation. Disease-specific interventions implicate macrophage metabolic pathways in inflammatory injury during ongoing MASH, while chromatin profiling identifies disease-associated regulatory changes [30,43,44]. Regression studies answer different questions: persistence of monocyte-derived Kupffer cells demonstrates a lasting compositional change [35], whereas increased macrophage collagen degradation identifies a restorative function [4]. These findings do not by themselves establish heightened inflammatory responsiveness to renewed exposure. The unresolved link is whether a defined regulatory program remains functionally consequential after injury attenuation and alters the response to a subsequent challenge. Testing that link requires distinguishing retained function in persisting cells from progenitor-derived replacement and continuing niche instruction.

### 6.6. Practical Requirements and Confounders in Testing Myeloid Memory in MASH

Dietary withdrawal should not be equated with removal of all relevant stimulation. Persistent fibrosis after clinical resolution of steatohepatitis documents incomplete structural recovery [91], while experimental MASH regression involves active macrophage-mediated matrix clearance [4]. Neither observation establishes the absence or presence of continuing inflammatory stimulation. Regression studies should, therefore, assess residual hepatocyte injury, lipid burden, and matrix state. Defective clearance of injured hepatocytes provides a potential source of continuing stimulation [85], whereas experiments linking matrix properties to macrophage activation or efferocytosis identify mechanical cues as another variable requiring assessment [102,103]. These mechanisms should be measured in the recovering liver rather than assumed to persist after dietary withdrawal.

Common culture assays reduce differences in extracellular exposure but may also change the population being tested. Environmental regulation of macrophage enhancer programs and reprogramming after tissue transfer demonstrate that cellular identity is not independent of the surrounding niche [33,34]. We, therefore, propose comparing freshly isolated and rested cells where feasible, with recovery intervals selected according to viability, basal activation, and identity retention. Residual intracellular cargo and drug carryover should also be assessed. Retention of an altered response under common conditions supports a cellular component but does not establish memory if stimulating material remains. Conversely, loss of the response could reflect either environmental dependence or culture-associated loss of the relevant program.

For paired human biopsies, assay scope should be matched to viable cell recovery. An initial study could prioritize a limited functional panel rather than require purification and extensive profiling of every macrophage subset. Population purity, viable cell numbers, and donor-level variability should be documented, and comparisons should use consistently defined populations. Human functional observations would be complemented by animal lineage tracing to resolve cellular origin and recruitment history.

Intervention timing introduces a further distinction. Recruitment blockade changes the hepatic macrophage compartment [76], whereas macrophage depletion can reduce scarring during established injury but impair matrix degradation during recovery [104]. Experiments should, therefore, separate program establishment, maintenance during regression, and effector activity during rechallenge. Reassessment after intervention withdrawal, alongside cell abundance and viability measurements, would help distinguish modification of a retained program from reduced recruitment, cell loss, or acute suppression of inflammatory output.

## 7. Conclusions

Improvement in body weight, glycemic control or hepatic fat content, histological resolution of steatohepatitis, and sustained protection against recurrence are distinct outcomes. Their relationship must be established longitudinally. Persistent inflammation may reflect continuing hepatocyte injury, residual lipotoxic signals, or an incompletely resolved fibrotic niche. Kupffer cell regulatory remodeling and replacement by monocyte-derived cells further complicate attribution to trained immunity [30,35]. The evidence supports the possibility that exposure history modifies hepatic inflammatory susceptibility alongside ongoing injury; whether this susceptibility persists after metabolic improvement and contributes to MASH recurrence remains unresolved.

Immunometabolic–epigenetic coupling provides a mechanistic basis for this proposition. Rested human monocyte models connect prior exposure to delayed cytokine responses, fumarate-associated histone methylation, and persistent histone lactylation [19,22,46]. These findings establish mechanisms in experimental training systems but do not establish their operation in hepatic macrophages after MASH regression. The unresolved causal link is whether a regulatory state established during MASH remains functionally consequential after the initiating injury subsides and increases the response to renewed injury.

Studies spanning injury, regression, and rechallenge should compare equivalent secondary insults while characterizing residual hepatic lipids, hepatocyte damage, and fibrosis. Lineage tracing and matched functional assays must distinguish responses in persisting Kupffer cells, recruited macrophages, and newly generated myeloid cells. Western diet experiments provide a precedent for persistence after dietary normalization and implicate hematopoietic progenitors [15]. Interventions should separately test state establishment, maintenance during regression, and inflammatory output during rechallenge. Human longitudinal studies should link metabolic and histological assessments to myeloid function; cross-sectional cellular profiles, circulating markers, and individual chromatin modifications cannot independently establish retained responsiveness or its contribution to recurrence.

If retained myeloid responsiveness contributes causally to MASH recurrence, its selective modification could complement treatment of continuing metabolic and tissue injury. Reduced cytokine production during treatment would not by itself demonstrate program resetting; reassessment after treatment withdrawal would help distinguish a durable functional change from acute suppression. Therapeutic evaluation should also measure whether lipid handling and repair are preserved, given the protective TREM2-dependent functions demonstrated during experimental MASH and regression [4,41]. The objective would be to reduce the excess inflammatory response attributable to previous disease without compromising host defense or tissue recovery. This remains a hypothesis to be tested through longitudinal and phase-specific interventions, rather than an established requirement for durable remission.

## Figures and Tables

**Table 2:** Demonstrated functions and unresolved memory-related questions for immunometabolic and epigenetic pathways.

Pathway or Regulatory Process	Demonstrated Function in the Cited Evidence	Disease and Cellular Context	Post-Withdrawal Evidence Relevant to Memory	Unresolved Question in MASH
F13A1-PKM2 and glycolytic regulation [43,110]	Controls macrophage metabolic activity and inflammatory injury	Experimental fatty liver/MASH and activated macrophages	Not established by the active disease interventions discussed	Does altered PKM2 regulation persist through regression and modify a later response?
Fumarate KDM5 [22]	Connects metabolite exposure to histone regulation and delayed cytokine responsiveness	Rested human monocyte training	Demonstrated in a defined comparator system	Does this coupling persist in hepatic macrophages after dietary withdrawal?
Succinate oxidation-mitochondrial ROS [111,112]	Supports selected inflammatory outputs during activation	LPS-activated macrophages	The cited activation experiments do not establish memory	Is continued metabolic flux required, or does a regulatory change remain after recovery?
IRG1-itaconate and NRF2-associated regulation [113,114]	Regulates tolerance or anti-inflammatory responses, depending on the experiment	Innate conditioning and activated macrophage systems	Temporal evidence is model-dependent; acute derivative treatment is not evidence of memory erasure	Does the pathway regulate activation, tolerance, or retained responsiveness during MASH regression?
TREM2-dependent lipid handling and repair [4,41,115]	Influences lipid-associated responses, inflammasome activity, and tissue repair	Hepatic macrophages, lipid-conditioning assays, and MASH models	Restorative function measured during MASH regression	Does a retained inflammatory response coexist with repair, and in which population?
LXR/AP-1-associated enhancer remodeling [30]	Alters Kupffer cells identity and disease-associated regulatory activity	Hepatic macrophages during experimental NASH	Functional persistence after regression not established	Which regulatory changes survive recovery and influence rechallenge?
ACLY-acetyl-CoA-histone acetylation [116,117]	Supports stimulus-dependent transcriptional programs	IL-4- or TLR4-stimulated macrophages	The cited experiments primarily address activation	Does phase-specific ACLY dependence extend to maintenance of hepatic myeloid memory?
PHGDH-NAD^+^-sirtuin regulation [95]	Links receptor expression and inflammasome processing to metabolic regulation	Inflammatory macrophages and endotoxin challenge	Post-regression hepatic persistence not established	Are transcriptional competence and cytokine processing durably altered after MASH improvement?
One-carbon metabolism SAM availability [118]	Supports methyl donor supply, H3K36me3, and inflammatory output	LPS-activated macrophages	Active stimulation dependence does not establish memory	Is methyl donor dependence retained during recovery, and does it govern a later response?
Histone lactylation [44,46,119,120]	Participates in late activation, rested training, and MASH-associated glycolytic feedback in different systems	Activated macrophages, BCG/β-glucan conditioning, and experimental MASH	Demonstrated in comparator training; not established through MASH regression–rechallenge	Which modification, locus, and cell population are required for a retained hepatic response?

## Data Availability

Data sharing is not applicable to this article as no datasets were generated or analyzed during the current study.

## Abbreviations

ACKR1, atypical chemokine receptor 1; ACLY, ATP citrate lyase; ACMSD, α-amino-β-carboxymuconate-ε-semialdehyde decarboxylase; ADAM17, a disintegrin and metalloproteinase 17; AP-1, activator protein 1; ASC, apoptosis-associated speck-like protein containing a CARD; ATAC-seq, assay for transposase-accessible chromatin using sequencing; BCG, bacillus Calmette–Guérin; BET, bromodomain and extraterminal domain; BMP, bone morphogenetic protein. CBP, CREB-binding protein; CCR2, C–C motif chemokine receptor 2; CCR5, C–C motif chemokine receptor 5; CD, cluster of differentiation; CITE-seq, cellular indexing of transcriptomes and epitopes by sequencing; CLEC4F, C-type lectin domain family 4 member F; CX3CR1, C–X3–C motif chemokine receptor 1; CXCL10, C–X–C motif chemokine ligand 10; DAMPs, damage-associated molecular patterns; DGAT2, diacylglycerol O-acyltransferase 2. DR5, death receptor 5; ECM, extracellular matrix; ER, endoplasmic reticulum; ERK, extracellular signal-regulated kinase; FGF21, fibroblast growth factor 21; GLS, glutaminase; GM-CSF, granulocyte–macrophage colony-stimulating factor; GPNMB, glycoprotein nonmetastatic melanoma protein B; HGF, hepatocyte growth factor; HIF-1α, hypoxia-inducible factor 1α. HK2, hexokinase 2; HSCs, hepatic stellate cells; HSPCs, hematopoietic stem and progenitor cells; IFN-γ, interferon γ; IGF1, insulin-like growth factor 1; IL, interleukin; IRE1α, inositol-requiring enzyme 1α; JNK, c-Jun N-terminal kinase; KDM5, lysine demethylase 5. KEAP1, Kelch-like ECH-associated protein 1; LAL, lysosomal acid lipase; LAMs, lipid-associated macrophages; LDH, lactate dehydrogenase; LDHA, lactate dehydrogenase A; LDL, low-density lipoprotein; LPS, lipopolysaccharide; LSECs, liver sinusoidal endothelial cells; LXR, liver X receptor; MASH, metabolic dysfunction-associated steatohepatitis. MASL, metabolic dysfunction-associated steatotic liver; MASLD, metabolic dysfunction-associated steatotic liver disease; MDH2, malate dehydrogenase 2; MITF, microphthalmia-associated transcription factor; MLK3, mixed lineage kinase 3; MS4A7, membrane spanning 4 domains subfamily A member 7; MSR1, macrophage scavenger receptor 1; mTOR, mechanistic target of rapamycin; mTORC1, mechanistic target of rapamycin complex 1; NAD^+^, nicotinamide adenine dinucleotide (oxidized form). NAFL, nonalcoholic fatty liver; NAFLD, nonalcoholic fatty liver disease; NASH, nonalcoholic steatohepatitis; NF-κB, nuclear factor κB; NLRP3, NLR family pyrin domain-containing 3; NOD2, nucleotide-binding oligomerization domain-containing protein 2; NRF2, nuclear factor erythroid 2-related factor 2; oxLDL, oxidized low-density lipoprotein; PAI-1, plasminogen activator inhibitor 1; PAMPs, pathogen-associated molecular patterns. PDGF, platelet-derived growth factor; PHGDH, phosphoglycerate dehydrogenase; PKM2, pyruvate kinase M2; ROCK1, Rho-associated coiled coil-containing protein kinase 1; ROS, reactive oxygen species; RUNX1, runt-related transcription factor 1; S1P, sphingosine-1-phosphate; SAM, S-adenosylmethionine; SAMs, scar-associated macrophages; SDHB, succinate dehydrogenase complex iron sulfur subunit B. SETD7, SET domain-containing lysine methyltransferase 7; SFRP2, secreted frizzled-related protein 2; SIRT, sirtuin; SPP1, secreted phosphoprotein 1; STING, stimulator of interferon genes; TCA, tricarboxylic acid; TGF-β, transforming growth factor β; TIM4, T-cell immunoglobulin and mucin domain-containing protein 4; TIMP, tissue inhibitor of metalloproteinases; TLR, Toll-like receptor. TNF, tumor necrosis factor; TRAIL, TNF-related apoptosis-inducing ligand; TREM2, triggering receptor expressed on myeloid cells 2; TRIF, TIR-domain-containing adaptor-inducing interferon-β; XBP1, X-box-binding protein 1; ZO-1, zonula occludens 1.
